# Strategies for Tobacco Control in India: A Systematic Review

**DOI:** 10.1371/journal.pone.0122610

**Published:** 2015-04-09

**Authors:** Ailsa J. McKay, Raju K. K. Patel, Azeem Majeed

**Affiliations:** 1 Department of Primary Care and Public Health, Imperial College London, London, United Kingdom; 2 London North West Healthcare NHS Trust, London, United Kingdom; University College London, UNITED KINGDOM

## Abstract

**Background:**

Tobacco control needs in India are large and complex. Evaluation of outcomes to date has been limited.

**Aim:**

To review the extent of tobacco control measures, and the outcomes of associated trialled interventions, in India.

**Methods:**

Information was identified via database searches, journal hand-searches, reference and citation searching, and contact with experts. Studies of any population resident in India were included. Studies where outcomes were not yet available, not directly related to tobacco use, or not specific to India, were excluded. Pre-tested proformas were used for data extraction and quality assessment. Studies with reliability concerns were excluded from some aspects of analysis. The Framework Convention on Tobacco Control (FCTC) was use as a framework for synthesis. Heterogeneity limited meta-analysis options. Synthesis was therefore predominantly narrative.

**Results:**

Additional to the Global Tobacco Surveillance System data, 80 studies were identified, 45 without reliability concerns. Most related to education (FCTC Article 12) and tobacco-use cessation (Article 14). They indicated widespread understanding of tobacco-related harm, but less knowledge about specific consequences of use. Healthcare professionals reported low confidence in cessation assistance, in keeping with low levels of training. Training for schoolteachers also appeared suboptimal. Educational and cessation assistance interventions demonstrated positive impact on tobacco use. Studies relating to smoke-free policies (Article 8), tobacco advertisements and availability (Articles 13 and 16) indicated increasingly widespread smoke-free policies, but persistence of high levels of SHS exposure, tobacco promotions and availability—including to minors. Data relating to taxation/pricing and packaging (Articles 6 and 11) were limited. We did not identify any studies of product regulation, alternative employment strategies, or illicit trade (Articles 9, 10, 15 and 17).

**Conclusions:**

Tobacco-use outcomes could be improved by school/community-based and adult education interventions, and cessation assistance, facilitated by training for health professionals and schoolteachers. Additional tobacco control measures should be assessed.

## Introduction

India is the second largest tobacco consumer, and third largest tobacco producer, in the world [1]. The current cost of tobacco use in India includes 1 million deaths per year (approximately 1/6 of all tobacco-related deaths worldwide), and billions of dollars of direct attributable health costs [2–4]. The problem is worsening, and by current trends, tobacco use will cause 13% of deaths in India by 2020 [4].

The variety of tobacco products used in India is greater than elsewhere, and associated with additional complications including a high burden of oral cancers from smokeless tobacco use [5]. The prevalences of diseases adversely affected by second hand smoke (SHS) exposure—in particular childhood respiratory infections and tuberculosis—are higher than in many parts of the world [6,7]. Various types of tobacco are grown in India; there are thousands of variously sized manufacturers regulated on several levels [8]; and there is a relatively large unregulated market [9]. The varied socio-cultural history and beliefs also has an impact, and there is complicated legislation addressing the various types of tobacco use, enforced to different extents at various administrative levels across the country [10].

The Government of India has become increasingly engaged with India’s tobacco problem over recent years. Some relatively small-scale preventative policies were introduced between 1975 and 2000 [11]. The more comprehensive Cigarette and Other Tobacco Products Act (COTPA; addressing tobacco use in public places, tobacco advertising, and sale and packaging regulations) was introduced in 2003, and the Framework Convention of Tobacco Control (FCTC) brought into force in 2005 [11]. This World Health Organization (WHO) treaty commits signatories to the implementation of wide-ranging measures to limit demand for tobacco, aid cessation of use, protect minors and non-users, regulate tobacco products, minimise the contraband market, and limit the negative influence of the tobacco industry [12]. It promotes various control strategies including pricing and taxation measures, smoke-free policies, tobacco product legislation, appropriate labelling of products (including health warnings), tobacco related education, prohibition of advertising and other promotion methods, provision of cessation programmes, control of illicit tobacco product trade, control of tobacco sale to/by minors, and support for alternative employment strategies for tobacco workers. Soon after committing to the FCTC, the Indian Government drew up a National Tobacco Control Programme to help achieve its provisions. The programme aimed to establish tobacco cessation centres, training programmes for teachers, health workers and others, educational interventions for schools and the general population, and mechanisms to monitor enforcement of tobacco control legislation, at the district level. State and national-level monitoring of these initiatives was also planned, as well as research activities regarding alternative livelihood options, establishment of tobacco product testing facilities and production of mass-media awareness campaigns [11]. A pilot has been undertaken, but the results, and information about the planned expansion of the programme, are not widely available [11]. Various aspects of this programme, and other control measures, have featured in the eleventh (2007–2012) and twelfth (2012–2017) Government of India Five Year Plans [13,14]. In line with the current Five Year Plan, the current Government has increased taxes on cigarettes, and announced plans for further strengthening of anti-tobacco legislation following a period of review.

Although the extent of any specific programmes designed to monitor and evaluate these recent wide-ranging interventions is unclear, standardised collection of both clinical and process outcomes has been achieved via the MPOWER (Monitor tobacco use and prevention policies, Protect people from tobacco smoke, Offer help to quit tobacco use, Warn about the dangers of tobacco, Enforce bans on tobacco advertising, promotion and sponsorship, Raise taxes on tobacco) and Global Tobacco Surveillance System (GTSS) frameworks, co-ordinated by the WHO and WHO/Centers for Disease Control and Prevention/Canadian Public Health Associations, respectively [12,15]. Both collect data regarding tobacco use prevalence, advertising, mass media campaigns, warnings on tobacco packaging and cessation services. The MPOWER outcomes provide additional information about monitoring of tobacco use prevalence and taxation, and the GTSS additional information about SHS exposure, school policies, and health professional training, among other things.

Some relatively idiosyncratic aspects of the Indian tobacco picture limit the ease of interpretation of some of these data. For example, there is an emphasis on smoking and cigarettes, with less information relating to other forms of tobacco use. The MPOWER outcomes are also reported at the national level, which limits their applicability in a country where there is subnational regulation of federal legislation, and where smaller-scale NGO-run anti-tobacco programmes appear to have achieved considerable success in particular districts/states [e.g. 16,17]. This is a particular issue because implementation of federal legislation has been troublesome—reportedly associated with a lack of awareness of the legislation, insufficient motivation, and workforce capacity challenges [e.g. 11,18].

Additional barriers to implementation of India’s national tobacco control strategies have included legal challenges by the tobacco industry, use of surrogate advertising methods and violations of some advertising regulations [11,18]. Thus despite the encouraging ongoing drive to improve tobacco control in India via wide-reaching means, these challenges encountered, and the absence of systematic evaluation of policy changes to date, together with an apparent idiosyncratic responsiveness to interventions applied elsewhere [e.g. 19,20], have resulted in ongoing uncertainty regarding the interventions likely to be most effective for India.

To help establish the extent of tobacco control in India, gauge the relative need for input into different aspects of control, and to establish so far as possible the extent to which different interventions in India have been successful, we performed a systematic review that aimed to answer the following questions:

1. To what extent are tobacco control measures evident in India?
2. Which tobacco control approaches implemented or trialled in India have been successful?

## Methods

**Fig 1:**
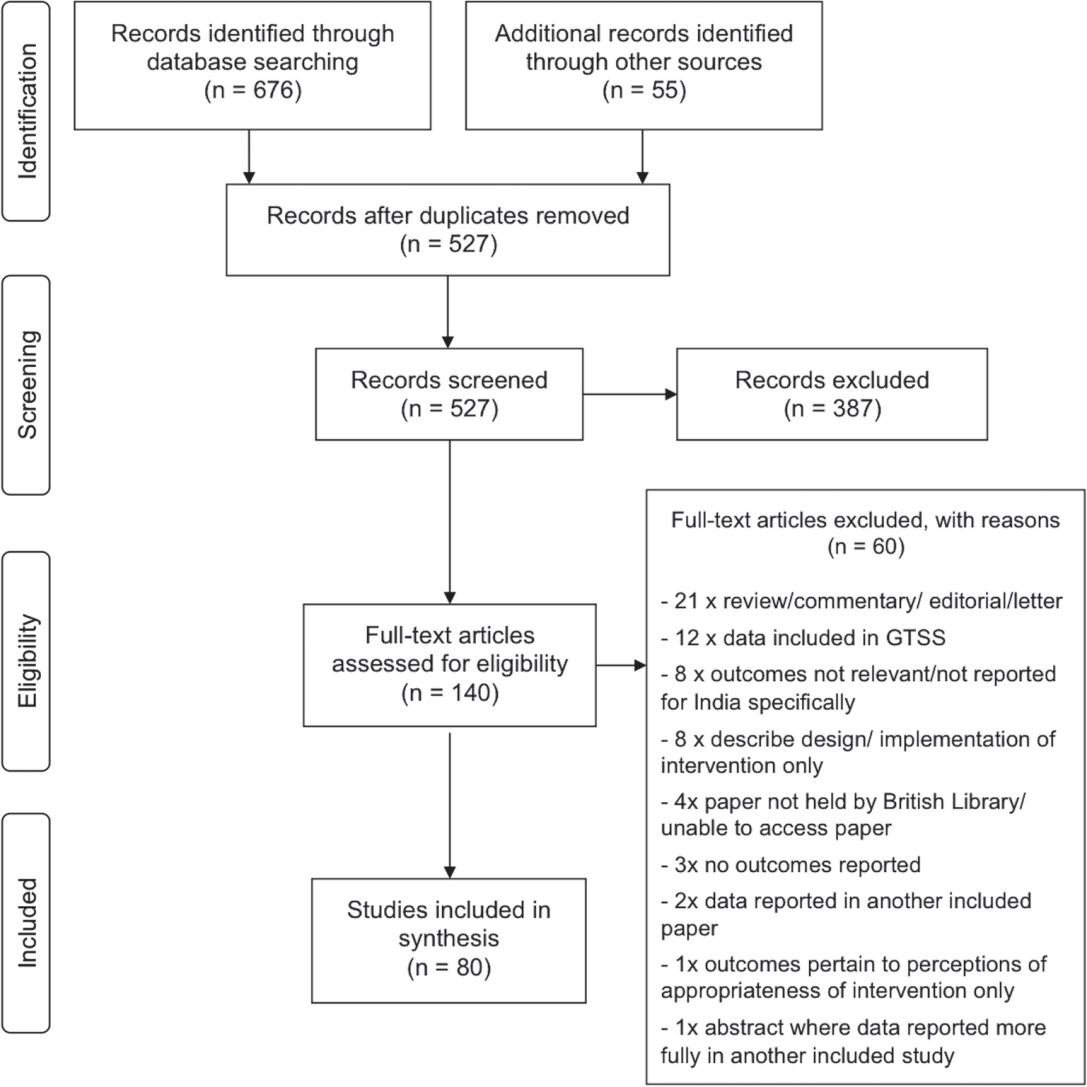
Flow chart demonstrating handling of papers returned by search. Chart adapted from: Moher D, *et al* ^21^

We followed the Preferred Reporting Items for Systematic Reviews and Meta-Analyses (PRISMA) Statement (see S1 PRISMA Checklist and Fig 1) [21].

### Ethical approval

Not required as this is a review article.

### Search

The review questions were used to formulate search terms (Table 1), and the search strategy was trialled to ensure that related well-cited articles and those identified in scoping searches would be returned. Adaptations were made where necessary, before final use. Databases (Medline, Embase, CENTRAL, PsychINFO, ERIC, Web of Science and CINAHL) were searched, using the terms in Table 1, on 23/9/12 (updated on 28/2/13 and 11/8/13). Lung India, Tobacco Control and the Indian Journal of Public Health were hand-searched on 29/9/12 (searches updated 17/3/13 and 11/8/13). No restrictions by date, language of publication, study design, publication type or publication status, were made. Reference lists of relevant papers were scanned, and we contacted local experts with the aim of identifying additional/unpublished data. The relevant national level data from the GTSS available on 1/9/13, were also identified. GTSS data are collected via one of four constituent questionnaire-based surveys: the Global Adult Tobacco Survey (GATS), Global Youth Tobacco Survey (GYTS), Global Health Professions Student Survey (GHPSS) or Global School Personnel Survey (GSPS) [15].

**Table 1:** Search terms.

	P	I	C	O	S
**Key terms**	All populations resident in India	Any	n/a	Tobacco	All
**Additional terms generated**	India	Prevention, preventive, preventative, cessation, strategy, treatment, management, therapy, campaign, approach, educat*, teaching, counselling, support, programme, program, ban, control, prohibit*, legislat*, law, statute, ordinance, bill, amendment, regulation, bupropion, amfebutamone, wellbutrin, zyban, elontril, patches, gum	n/a	Smoking, tobacco, nicotine, cigarette, pipe, beedi, bedi, bidi, khaini, paan, gul, gutkha, ‘pan masala’	All

PICOS identifiers from research questions (‘key terms’) and database- and thesaurus- derived alternatives (‘additional terms’) used to generate database searches. Stars indicate where all database terms based on the attached stem were included. Terms within each column were distinguished using the OR function and the terms in differing columns combined using AND.

### Selection

We included studies of any population resident in India, any form of tobacco use, any type of intervention and any process or clinical outcome measure. Indeed the outcomes monitored were of interest *per se*. Studies that were descriptions of interventions not yet implemented or currently in trial, or of methods for intervention development, were excluded. Those where outcomes were not directly related to tobacco use, not specific to India, or limited to perceived appropriateness of interventions, were also excluded. We did not include commentaries, news articles, reviews, letters, or other opinion pieces unless new syntheses were included, and did not include studies where the contained data were reported in entirety in another included study. Studies reporting data collected for the GTSS were only included if the data were not already available as part of the national GTSS results for India, to avoid inclusion of data in replicate.

One reviewer initially assessed study eligibility by screening titles/abstracts. Studies accepted at this point were reviewed more fully, and any further exclusions again made according to the above criteria. All remaining studies progressed to data extraction.

### Data extraction

Basic study data were extracted using a proforma based on the checklist recommended by the Centre for Reviews and Dissemination, York [22]. It was pre-tested on a subset of papers, and changes made as necessary before final use. The broad headings under which data were collected are listed in Table 2. One reviewer used the proforma for initial data collection. A second reviewer checked the collected data, and any discrepancies were resolved by re-referral to the study and consensus decision.

**Table 2:** Data extraction and quality assessment checklists.

General data extraction	Quality assessment checklist
∙ Study dates (or publication date if not available)	1 Type of report (e.g. published/unpublished, whether or not subject to peer review, study potentially in progress (e.g. conference proceedings), or completed)
∙ Study design	2 Clear aims/objectives
∙ Type of report	3 Clear and appropriate methods, including sampling/recruitment (4), inclusion/exclusion criteria (5), and data collection
∙ Number of participants (enrolled, excluded and lost to follow up)	6 Appropriate and rigorous analysis
∙ Participant characteristics (including age, sex, tobacco-use status, socioeconomic status and professional group, where available)	7 Outcomes not reported, or additional outcomes reported
∙ Study setting (location, and urban or rural)	8 Risk of bias in selection
∙ Definition of diagnosis used	9 Risk of bias in measurement and outcomes
∙ Measurement/assessment tool	10 Limitations discussed
∙ Outcomes (including subgroup data for age, sex, urban/rural residence, tobacco-use status and professional group, where available)	11 Funding information and information regarding conflicts of interest

The numbers beside the quality assessment criteria are used to indicate how quality for each criterion has been rated, in Tables 4–7

### Quality assessment

Data for quality assessment were extracted using a second proforma. Our quality checklist includes the ‘component ratings’ of the Effective Public Health Practice Project Quality Assessment Tool [23] and bias assessment recommended by the Cochrane Handbook [24]. Again the proforma was pre-tested before use. Data were independently collected by two reviewers, who then independently rated each quality domain (as listed in Table 2) as ‘satisfactory’, ‘not satisfactory’, or ‘not assessable’. The main reasons for any concern about reliability of results were also noted. Inconsistencies in ratings were resolved by consensus decision (initial kappa agreement (standard error) = 0.86 (0.06)). Where the number of ‘unsatisfactory’/‘not-assessable’ ratings exceeded eight, the study was omitted from some aspects of analysis (see below) due to concerns about the reliability of reported outcomes. Where the number of ‘unsatisfactory’/‘not-assessable’ ratings fell between six and eight the main concerns about the study were considered and independent decisions about inclusion/exclusion made by two reviewers. An independent decision from a third reviewer was sought in cases of disagreement.

### Data synthesis

An initial assessment of the number of studies identified, and time-trends in production and quality, was made. The studies were then divided into two subsets for further analysis:

1. Studies describing the extent of tobacco control measures in India
2. Studies relating to trialled interventions

In view of the heterogeneity in study design and outcomes investigated in both categories, a narrative synthesis was initially undertaken. Summaries of both sets of data were produced—by subcategories corresponding to the articles of the FCTC. Where the articles pertained to a broad range of studies (articles 12 and 14), studies were grouped by theme: population implicated (young people/adults/healthcare professionals) in the case of Article 12; population surveyed (healthcare professionals, trainees, and patients) in the case of Article 14.

The nature of study aims and outcomes were examined. Studies with identified reliability concerns were then excluded before assessment of outcomes and an examination of each category of studies to investigate the possibility of differences by subgroup (age, sex, urban/rural location, tobacco use status, or professional group, as applicable—all identified *a priori*). Proposals were formed based on general initial analysis outcomes, and inconsistencies examined.

The extent of clinical and methodological diversity limited the scope for meta-analysis of the studies of trialled interventions. Although three studies of similar school-based interventions were identified, different types of effect estimate were reported for each. We were therefore unable to pool these, and we were unable to obtain the underlying data. The only other trial sub-category containing multiple studies pertained to non-pharmacological cessation interventions. Meta-analysis was performed using a random effect model, for unadjusted and adjusted intention to treat data. Estimated summary odds ratios, confidence intervals and measures of heterogeneity were produced. Calculations were performed using RevMan version 5.3 (Copenhagen: The Nordic Cochrane Centre, The Cochrane Collaboration, 2014).

## Results

Additional to the GTSS data, the search yielded 527 studies for review. The processing of search results is detailed in Fig 1. Eighty studies were considered suitable for inclusion. Other than five studies from the late 1980s/early 1990s, all had been published since 2002, and half since 2010. The studies excluded from some aspects of synthesis due to reliability concerns (see Methods), included 21/40 pre-2010, and 18/40 post-2009, studies. Some components of a further four studies (two pre-2010) were similarly excluded from some aspects of synthesis.

Among the studies identified, 56 described various aspects of tobacco control to date, and 25 the results of trialled interventions (one study achieved both). Table 3 lists the articles of the FCTC relating to the reduction of demand and supply of tobacco, and the number of identified studies in each category (trial/non-trial) addressing each. The majority of studies pertained to articles 12 (10 trial and 38 non-trial studies) and 14 (10 trial, and 17 non-trial). Smaller numbers of studies relating to articles 6, 8, 11, 13 and 16 were also identified. The studies included in all aspects of synthesis are summarised, by FCTC article, in Tables 4 to 7 (those excluded from analysis of outcomes summarised in Tables A to E in S1 File), and discussed further—by FCTC article—below.

**Table 3:** Summary of studies identified by FCTC Article.

FCTC article	Included pre-2005	Included post-2005	Total included	Excluded pre-2005	Excluded post-2005	Total excluded	GATS (2009)	GYTS (2006, 2009)	GHPSS (2005, 2009—medical and dental students; 2007—nursing students; 2008—pharmacy students	GSPS (2006, 2009)
**6**		1	1		1	1				
**7**										
**8**	1	4	5	3	7	10	✓	✓	✓	✓
**9**										
**10**										
**11**		1	1		6	6	✓			
**12**	12	21	33	6	11	17	✓	✓	✓	✓
**13**	3	1	4		5	5	✓	✓		
**14**	4	14	18	2	8	10	✓		✓	
**15**										
**16**	1	1	2		4	4		✓		
**17**										

The numbers of identified studies included and excluded from full analysis are displayed by FCTC Article. The number of studies performed before and after 2005 (year FCTC brought into force) are also shown. Studies that involved data collection both pre- and post- FCTC have been listed as ‘post-2005’. Where the dates of the study were not reported, the ‘pre-/post- 2005’ designation was applied according to the date of publication. Five identified studies with outcomes that relate only to multiple articles of the FCTC are not included in the table. All were excluded from full analysis. The table also demonstrates which articles of the FCTC the data collected for the Global Tobacco Surveillance System relate to, and the years in which these data were collected.

**Table 4:** Studies related to FCTC articles 6, 8, 11, 13 and 16.

Related FCTC Article	Ref. (pub. year)	Study dates & location	Sample size & characteristics	Tobacco use prevalence	Methods	Main outcome measures (bold) & results	Quality assessment (numerical ratings and main concerns)
**6 (Price and tax measures to reduce the demand for tobacco)**	25 (2012)	2011; Jaipur (U)	n = 25 shopkeepers, 500 tobacco users; > 18 years; sex NR	NR for shopkeepers, otherwise 100% tobacco users	Questionnaire < 4 weeks post-tax rise	**Post tax rise: cigarettes: price change:** + 19%*, **sales:**- 14%^#^, **consumption**:- 15%*; **bidis: price**: +21%*, **sale:** -23%*, **consumption:**- 13%*; chewing tobacco: price: + 68%^#^, **sale:**- 38%**, **consumption:**- 21%* (*p < 0.0001; **p < 0.001; ^#^p< 0.05)	SA: 1, 3–5, 8, 11; US: 2, 7, 10; NA: 6, 9 (Small n-numbers, limited follow-up, unclear when data collected, possible recall bias, conclusions do not all follow from data)
**8 (Protection from exposure to tobacco smoke)**	26 (2013)	Dates NR; Karnataka (U/R NR)	n = 456, final year dental students, 30.5% male, mean age 22.7±0.94 years	9.1% current users, 1.3% former users	Self-administered questionnaire	**Smoking prohibited on campus**: 86.5%;	SA: 1–6,8; US: 10,11; NA: 7,9 (Data collected unclear, possible selective reporting)
**8**	27 (2011)	2009; Karnataka (U/R NR)	n = 329; dental students of 3 colleges in Karnataka; 20–26 years; 29.7% male	7% current smokers; 5% ex-smokers	Questionnaire (delivery method unclear)	**Aware smoking prohibited** in different areas of campus: 91.2%- 98.2% (variation by type of area)	SA: 1, 2, 4–7, 9; US: 8, 10, 11; NA: 3 (Convenience sample, multiple comparisons)
**8**	28 (2012)	2005 & 2009; all India (U+R)	Medical/dental students: n = 1176 (2005), 1523 (2009)/ 1339 (2005), 711 (2009); age, sex NR	Medical/dental students: smokers: 13.4%/6.5%; smokeless users: 11.6%/8.6%	Secondary analysis of GHPSS data	Between 2005–09, S reduction in **exposure to SHS at home** (from mean 56.4% to 40.0%) **and in public places** (mean 68.4% to 52.5%) among dental students. No change among medical students. No **change in proportion of students reporting school smoking ban**	SA: 1, 3–9, 11; US: 10; NA: 2
**8**	29 (2008)	2003 & 2006; all India (U+R)	n = 68077 (2003), 12086 (2006); 13–15 years; sex NR	2003/2006: ever smokers, 9.5%/12.0%; current smokers: 4.2%/3.8%; other tobacco users: 13.6%/11.9%	Secondary analysis of GYTS data	Between 2003–06: S reduction in SHS exposure at home (mean 36.4% to 26.6%) & in public places (mean 48.7% to 40.3%)	SA: 1–5, 7–10; US: 11; NA: 6 (Little demographic information reported, natural experiment/ no controls)
**8**	30 (2004)	2004; Bihar (U/R NR)	n = 521 doctors; 55.3% GPs; 82% 25–55 years; 89.3% male	Cigarette smokers: 7%; ‘other tobacco users’: < 1%; `chewing/ applied product’ users: 11.7%	Self-administered questionnaire	**Smoking prohibited across campus/in enclosed spaces/in public areas**: 25.3%/10.8%/10.4%; **smoking permitted only in designated areas**: 2.4%; **no smoke-free policy**: 49.1%	SA: 1, 3, 4, 8, 11; US: 2, 9, 10; NA: 5–7 (Aims unclear, data collected & analysis intentions NR, some reported outcomes ambiguous)
**11 (Packaging and labelling of products)**	31 (2011)	Dates NR; Wardha, Maharashtra (R)	n = 242; adolescents of 6 ‘tribal villages’; 11–19 years; 33.9% female	Current users: 52.1%	Interview	**Aware of warnings on packs:** 62/242; **able to interpret warnings**: 20.97%	SA: 1, 2, 6, 10; US: 11; NA: 3–5, 7–9 (Recruitment & extent of data collection unclear, risk of recall bias)
**13 (Tobacco advertising, promotion and sponsorship)**	53 (2011)	2009; New Delhi (U)	n = 3956; school students; 12–16 years; 54.1% male	Ever use: 5.3%	Self-administered questionnaire	**Mean** 60% (31/59 items) of list of **movies depicting tobacco use viewed**; 7.3% **owned tobacco branded item**	SA: 1, 3–8, 10, 11; US: 2; NA: 9 (1/3 schools consented to participate, possible recall bias)
**13**	29 (2008)	2003 & 2006; all India (U+R)	n = 68077 (2003), 12086 (2006); 13–15 years; sex NR	2003/2006: ever smokers, 9.5%/12.0%; current smokers: 4.2%/3.8%; other tobacco users: 13.6%/11.9%	Secondary analysis of GYTS data	Between 2003–2006: NS difference in exposure to advertisements, S increase free cigarette offers from tobacco companies (mean 8.0% in 2003, 11.2% in 2006)	SA: 1–5, 7–10; US: 11; NA: 6 (Little demographic information reported, natural experiment/no controls)
**13**	54 (2008)	2004; Delhi & Chennai (U)	n = 11642; school students; mean age 11.2 years (6^th^ graders), 12.9 years (8^th^ graders); 54.9% male	NR	Self-administered questionnaire	**Reported favourite advertisement**: 493; r**ecalled brand names:** 238; had **seen advertisements in > 4**/**1–4/none of places listed in survey:** 37%/50%/13.2%	SA: 1–4, 6–9, 11; US: 10; NA: 5 (Multiple comparisons, possible recall bias)
**13**	55 (2004)	2000; Bihar; 59.8% R	n = 2636 school students; 13–15 years; 76% male	Ever users: 71.8%; current users: 58.9%	Self-administered questionnaire	**Gutka/cigarette advertisements in media seen** by ‘almost all’;	SA: 1–5, 8; US: 10, 11; NA: 6, 7, 9 (Data collected & intended analysis unclear, some reported outcomes unclear)
**16 (Sale to and by minors)**	29 (2008)	2003 & 2006; all India (U+R)	n = 68077 (2003), 12086 (2006); 13–15 years; sex NR	2003/2006: ever smokers, 9.5%/12.0%; current smokers: 4.2%/3.8%; other tobacco users: 13.6%/11.9%	Secondary analysis of GYTS data	Between 2003–06: NS difference in proportion of users purchasing cigarettes in a store (mean 65.9% in 2003, 51.9% in 2006)	SA: 1–5, 7–10; US: 11; NA: 6 (Little demographic information reported, natural experiment/no controls)
**16**	55 (2004)	2000; Bihar; 59.8% R	n = 2636 school students; 13–15 years; 76% male	Ever users: 71.8%; current users: 58.9%	Self-administered questionnaire	**Bought tobacco in store**: 56.1%; **not refused purchase due to age**: 77.2%	SA: 1–5, 8; US: 10, 11; NA: 6, 7, 9 (Data collected & intended analysis unclear, some reported outcomes unclear)

Reviewed studies relating to FCTC articles 6, 8, 11, 13 and 16 included in all aspects of synthesis. The numbers following the different quality categories (SA, US, NA) indicate the aspect of quality assessment (see Table 2) rated as satisfactory (SA), unsatisfactory (US) or not-assessable (NA). All studies were of cross sectional design, or secondary analyses of cross-sectional surveys. U = urban; R = rural; NR = not reported; GHPSS: Global Health Professions Student Survey; S = significant; NS = non-significant; SHS = second-hand smoke; GYTS = Global Youth Tobacco Survey

**Table 5:** Studies related to FCTC Article 12: Education, communication, training and public awareness.

Population implicated	Ref (pub. year)	Study dates & location	Sample size & characteristics	Tobacco use prevalence	Methods	Main outcome measures (bold) & summary results	Quality assessment (numerical ratings and main concerns)
**Young people**	31 (2011)	Dates NR; Maharashtra (R)	n = 242; adolescents of 6 ‘tribal villages’; 11–19 years; 66.1% male	52.1%	Interview	94.2% **aware of risks of tobacco use**; most have incomplete/inaccurate knowledge	SA: 1, 2, 6, 10; US: 8, 9; NA: 3–5, 7, 11 (Recruitment & extent of data collection unclear, risk of recall bias)
**Young people**	32 (2008)	2008; Wardha (R)	n = 385 in survey; 15–19 years; 47.5% male	Smokers: 39% (68.3% boys, 12.4% girls)	Interview	**Snuff used for cleaning teeth**: 25.6% males, 72% females; would *not* **consider tobacco as medication**: 3%; **aware of link with cancer**: 61.8%, with **other diseases:** 0.8–38.2%; **aware addictive:** 10.1%.	SA: 1–4, 8, 11; US: 7, 9, 10; NA: 5, 6 (Extent of data collection & intended analysis unclear, multiple comparisons)
**Young people**	33 (2005)	Dates NR; Maharashtra & Bihar (U+R)	Maharashtra/Bihar: n = 954/524; 74.2%/78.6% male; school teachers; age NR;	Current tobacco users: 30.5% (Maharashtra), 77.8% (Bihar)	Secondary analysis of GSPS data	Maharashtra/Bihar: **Students taught about: short-term health effects of tobacco**: 77.4%/0.6%, **long-term effects**: 74.7%/0.5%; **prevalence of youth use**: 40.3%/0.1%; **communication skills**: 54.2%/0.2%; **goal setting**: 23.5%/0.2%; **peer pressure**: 37.4%/0.8% (Differences in all outcomes between the 2 regions; p <0.01 in each case)	SA: 1–9; NS: 10, 11; NA: n/a (Multiple comparisons)
**Young people**	34 (2004)	2000 (state schools), 2001 (federal schools); Bihar (U+R)	n = 2636 state school students, 3951 federal school students; 13–15 years; sex NR	Ever tobacco use: 72.8% (R state schools), 35.6% (R federal), 70.0% (U state), 35.2% (U federal)	Secondary analysis of GYTS data	Students in federal schools S more **teaching on dangers of smoking** than those in state schools (72.7±4.7% (R) & 51.6±3.7% (U) cf. 1.8±1.5% (R) & 2.5±2.8% (U)). Students in federal schools S more teachings on **reasons why individuals of their age smoke** (49.9±4.4% (R) & 37.6±2.8% (U), cf. 0±0% (R) & 1.9±2.4% (U))	SA: 1, 3–5, 8; US: 2, 10, 11; NA: 6, 7, 9 (Aims of data collection & analysis NR, no statistics reported)
**Young people**	35 (2004)	2000, Goa (U/R NR)	n = 2256; school students; 13–15 years; 56% male	Current users: 4.5%; ever users: 13.5%	Self-administered questionnaire	Non-users/current users: **tobacco helps relieve toothache, morning motion**: 19.2%/38.1%, **smoking harmful**: 62.9%/29.1% **smokeless use harmful**: 62.1%/22.4%; **SHS harmful**: 59.2%/41.3%	SA: 1, 3, 8; US: 2, 7, 10; NA: 4–6, 9, 11 (Aims unclear, data collection & analysis intensions NR, some results ambiguous)
**Adults**	36 (2012)	2009–10; all India; ‘majority R’	n = 2898; 16–50 years; 32% female; access to mass media	100% smokeless only & dual tobacco users	Interview in month following 6-week GOI television/radio campaign targeting smokeless users	65% **campaign aware**; campaign led 75–77% to feel **concern about tobacco use**; 26–41% **to encourage quitting**; Smokeless-only users: **S higher knowledge scores** (p≤0.05), & **cessation-oriented behaviours** (p ≤ 0.001) among campaign-aware cf. non-aware; Dual users: awareness not associated with **cessation attempts**; unaware self-report **use of less tobacco** post-campaign	SA: 1–11; US: n/a; NA: n/a (Retrospective, no control group/pre-intervention survey, possible recall bias)
**Adults**	37 (2009)	2006–07; Kerala (U)	n = 100; patients with diabetes; mean age 55.8±11.9 years; 100% male	100% smokers	Interview	**Smoking doesn’t influence/’mildly aggravates’/’very much aggravates’ diabetes**: 52%/13%/35%; **1–5 sticks/day safe**: 34%/30% (cigarette/bidi smokers); **6–25 sticks/day does not cause ‘too much harm’**: 34%/46% (cigarette/bidi smokers)	SA: 1, 2, 4, 8, 11; US: 10; NA: 3, 5–7, 9 (Interview methods, interview & intended analyses unclear content)
**Adults**	38 (2012)	2006; Maharashtra & Bihar (U+R)	n = 249; smokers; > 18 years; 74.3% male	100% (smokers)	Interview	79.4% consider **smoking ‘not good’ for health**: ‘majority’ **feel smoking not harming them**, 44.4% **believe tobacco has not harmed their health**	SA: 1–4, 6–8, 10, 11; US: n/a; NA: 5, 9 (Inclusion/exclusion criteria NR, relatively ambiguous outcomes analysed)
**Adults**	39 (2011)	2006; Maharashtra & Bihar (U+R)	n = 248; smokeless tobacco users; > 18 years; sex NR	100% (smokeless users)	Interview	Bihar/Maharashtra: **smokeless tobacco**: **‘not good’ for health**: 71.6%/64%; has **not harmed health**: 44%/71.9%; **causes mouth cancer** 87.3%/64.9%; **causes gum disease**: 69.4%/62.3%; **causes difficulty opening mouth**: 57.5%/53.5%	SA: 1,2,4,11; US: 6,7,10; NA: 3,5,8,9 (Sampling & intended data collection unclear, demographic outcomes for smokeless users NR, multiple comparisons)
**Adults**	31 (2011)	Dates NR; Wardha, Maharashtra (R)	n = 242; adolescents of 6 ‘tribal villages’; 11–19 years; 33.9% female	Current users: 52.1%	Interview	**Heard prevention message**: 69% (73.5% via radio, 44.3% via television); **able to interpret message**: 3/167	SA: 1, 2, 6, 10; US: 11; NA: 3–5, 7–9 (Recruitment & extent of data collection unclear, risk of recall bias)
**Adults**	40 (2010)	Dates NR; Assam (U)	n = 300; mean age 18–80 years; 52.3% male	63.7% ever users; 52.3% current users (32.0% smokers, 29.3% smokeless users)	Interview	97.3% **aware of tobacco-related health problems; aware of link with cancer:** 53.0%, with **other diseases**: 1.3–36.7%; **aware SHS harmful**: 78.7%; 45.7% ‘good’ **awareness of COTPA and main provisions**	SA: 1,2,4,5,7,9–11; US: n/a; NA: 3,6,8 (Sampling methods unclear)
**Adults**	41 (2006)	Dates NR; Nagpur City (U)	n = 1168, mean age: males: 34.2±2.1 years, females: 33.7±3.8 years; 50.5% male	Females/males: smokeless use: 12.6%/30.8% smokers: 0%/63%	Interview	**Smoking considered harmful to health**: 82.8%; **smokeless users concerned about own** health: 23.9%; believe **tobacco use ‘keeps bowel habits normal’**: 62.4%	SA: 2,4,5,8; US: 1,10,11; NA: 3,6,7,9 (Little information regarding methods, area sampled, data collected & intended analysis; no discussion of results)
**Adults**	42 (2004)	2002; Rajasthan (U/R NR)	n = 909; school personnel; 61.7% < 40 years, 1% > 60 years; 69% male	Ever users: 35.9%; current users: 14.4%	Self-administered questionnaire	**Tobacco considered addictive:** 42.2%/55.3% ever-/never-tobacco users; considered **cause of serious diseases:** 78.4%, consider **SHS exposure harmful**: 84%	SA: 1–5,8; US: 10,11; NA: 6,7,9 (Data collected & intended analysis unclear)
**Adults**	43 (2004)	2001; Orissa (U/R NR)	n = 517; school personnel; ages: < 40–59 years; 82.9% male	Smokers: 18.3%/16.6% (cigarettes/bidis); smokeless users: 24.2%	Self-administered questionnaire	**Tobacco considered addictive:** 90.7%; c**onsidered to have serious health consequences**: 94.2%; **SHS harmful**: 90.8%	SA: 1,3,4,8; US: 2,7,10, 11; NA: 5, 6, 9 (Aims of data collection NR, intended analysis unclear, results of comparisons NR)
**Adults**	44 (2004)	2001; West Bengal (U/R NR)	n = 663; school personnel; < 40 to > 60 years; 68.5% male	Smokers: 30.9%; smokeless users: 13.1%	Self-administered questionnaire	**Tobacco considered addictive:** 88.6%; **considered to have serious health consequences**: 88.6%; **SHS harmful**: 92.3%	SA: 1,3,4,8; US: 2,7,10, 11; NA: 5, 6, 9 (Aims of data collection NR, intended analysis unclear)
**Adults**	45 (2004)	2001; Uttar Pradesh (UP) & Uttaranchal (Ut) (U/R NR)	n = 993 (UP); 705 (Ut); school personnel; < 40– >60 years; UP 92% male, Ut 84.1% male	UP/Ut: smokers: 27.3%/23.7% cigarettes, 17.5%/13.1% bidis; smokeless users: 21.9%/29.2%	Self-administered questionnaire	UP/Ut: **Tobacco considered addictive:** 84.5%/69%; **considered to have serious health consequences**: 88.9%/66.4%; **SHS harmful**: 88.5%/70.6%	SA:1,3,4,8; US: 2,7,10, 11; NA: 5, 6, 9 (Aims of data collection & intended analysis unclear)
**Healthcare professionals**	46 (2013)	2011–12; Thrissur (U)	n = 637, age & sex NR; dental students	NR	Self-administered questionnaire	97.6% **aware of link with oral cancer**, 45.1% **of link with implant failure**	SA: 1–3,6,7,9,10; US: 4,8,11; NA: 5 (Convenience sample, inclusion/exclusion criteria NR)
**Healthcare professionals**	47 (2013)	2011; Andhra Pradesh (AP) & Gujarat (‘primarily R’)	n = 238; 82.2% GPs, 17.8% alternative health practitioners; Gujarat/AP: mean ages: 32.2±7.7/ 36.4±8 years; 79.6%/63.1% male	Ever users: 10.4%; current users: 3.9%	Interview	**Knowledge of effective counselling**: 92.2%; **knowledge of NRT**: 66.1%; **sufficient background to provide cessation services**: 17%	SA: 1–5,8,11; US: 7,10; NA: 6,9 (Limited information regarding analysis)
**Healthcare professionals**	48 (2013)	2009–10; Kerala & Karnataka (U/R NR)	Medical faculty/ students: n = 713/2585; mean age 32.9±9.7/20.3±1.7 years, 59%/47.7% male	28% faculty, 26% students ever smokers	(a) curriculum review; (b) self-administered questionnaire	**Curriculum review**: tobacco not mandated part of any curriculum; information delivered not systematic/sufficient; Student survey: **tobacco-related teaching** in class: 64%; teaching ‘minimal’ & not examined; agree **no safe level of smoking**: 54%; Faculty survey: agree **no safe level of smoking**: 89%; **sufficient experience to aid cessation:** 20%	SA: 1–8, 10, 11; US: n/a; NA: 9 (College selection, analysis intentions & classification of some variables unclear)
**Healthcare professionals**	26 (2013)	Dates NR; Karnataka (U/R NR)	n = 456, final year dental students, 30.5% male, mean age 22.7±0.94 years	9.1% current users, 1.3% former users	Self-administered questionnaire	**Received teaching regarding link with cancer**: 100%	SA: 1–6,8; US: 10,11; NA: 7,9 (Data collected unclear, possible selective reporting)
**Healthcare professionals**	27 (2011)	2009; Karnataka (U/R NR)	n = 329; dental students of 3 colleges in Karnataka; 20–26 years; 29.7% male	7% current smokers; 5% ex-smokers	Questionnaire (delivery method unclear)	**Received teaching regarding link with cancer: 97.2%**	SA: 1, 2, 4–7, 9; US: 8, 10, 11; NA: 3 (Convenience sample, multiple comparisons)
**Healthcare professionals**	49 (2011)	2007; Bangalore (U)	n = 76; clinical residents; mean age 28±2.9 years; 68% male	NR	Self-administered questionnaire	> 2/3 **unaware of smoking prevalence in India**, 20% suggest **tobacco use not linked to stroke**, 25% **not aware of NRT**; 25% **considered tobacco use a permissible 'fun activity' not to intervene with**	SA: 1–4,7–10, 11; US: n/a; NA: 5, 6 (Small n-number, questionnaire components NR)
**Healthcare professionals**	30 (2004)	2004; Bihar (U/R NR)	n = 521 doctors; 55.3% GPs; 82% 25–55 years; 89.3% male	Cigarette smokers: 7%; ‘other tobacco users’: < 1%; `chewing/applied products’: 11.7%	Self-administered questionnaire	**Tobacco use not considered safe in any form/amount:** 90.4%	SA: 1,3,4,8,11; US: 2,9,10; NA: 5–7 (Aims unclear, data collected & analysis intentions NR, some reported outcomes ambiguous)
**Healthcare professionals**	50 (2009)	2003; Kerala (U & semi-U)	n = 110 male faculty, 154 male doctors, 75 female doctors; mean age 42.2±7.7 years	63.1% never smokers	Self-administered questionnaire	5 **cigarettes/day considered harmful** by faculty and female doctors, 3 by male doctors; **smoking > 6/day harmful**: 1/4 faculty, 1/3 doctors; **1/day harmful**: 42% faculty, 68% male doctors, 17% female doctors	SA: 1–4, 8; US: 7, 9–11; NA: 5,6 (Extent of data collection & analysis unclear, low participation rates in some groups)
**Healthcare professionals**	51 (1995)	Dates NR; Punjab (U & ‘semi-U’)	n = 106; private GPs; age NR; 90.6% male	7.5% smokers	Self-administered questionnaire	11.3% **aware of cessation clinics**, 9.4% **aware of low tar cigarettes**, 5.7% **aware of nicotine gum**	SA: 1–4, 8; US: 9–11; NA: 5–7 (Data collection methods & data collected NR, no analysis reported)
**Healthcare professionals**	52 (1991)	Dates NR; Mumbai (U)	n = 363; GPs; age NR; 85.4% male	Smokers: 7.7%; smokeless users: 8.7%	Self-administered questionnaire	97% **considered smoking and chewing harmful**; 15% **considered bidis less harmful,** 15% **more harmful, than cigarettes**	SA: 1–4, 8; US: 7, 9–11; NA: 5,6 (Data collection methods, data collected & intended analysis unclear)

Reviewed studies relating to FCTC Article 12 included in all aspects of synthesis, sub-categorised by population implicated. The numbers following the different quality categories (SA, US, NA) indicate the aspect of quality assessment (see Table 2), rated as satisfactory (SA), unsatisfactory (US) or not-assessable (NA). All studies were of cross-sectional design, or secondary analyses of cross-sectional surveys. NR = not reported; U = urban; R = rural; NR = not reported; GSPS = Global School Personnel Survey; GYTS = Global Youth Tobacco Survey; S: significant; NS: non-significant; SHS = second hand smoke; COTPA: Cigarettes and Other Tobacco Products Act; GOI = Government of India; GP = general practitioner; NRT = nicotine replacement therapy

**Table 6:** Article 14: Demand reduction measures concerning tobacco dependence and cessation.

Population surveyed	Ref. (pub. year)	Study dates & location	Sample size & characteristics	Tobacco use prevalence	Methods	Main outcome measures (bold) & results	Quality assessment (numerical ratings and main concerns)
**Medical/ dental practitioners**	47 (2013)	2011; Andhra Pradesh (AP) & Gujarat (‘primarily R’)	n = 238; 82.2% GPs, 17.8% alternative health practitioners; Gujarat/AP: mean ages: 32.2±7.7/ 36.4±8 years; 79.6%/63.1% male	Ever users: 10.4%; current users: 3.9%	Interview	**Received training in medical school/at work**: 29%/16.5%; **have knowledge of effective counselling**: 92.2%; **feel background sufficient to deliver cessation services**: 17%	SA: 1–5,8,11; US: 7,10; NA: 6,9 (Limited information regarding analysis)
**Medical/ dental practitioners**	48 (2013)	2009–10; Kerala & Karnataka (U/R NR)	n = 713; medical faculty; mean age 32.9 ± 9.7 years, 59% male	28% ever smokers	Self-administered questionnaire	**Perceived sufficient experience to help patients quit**: 20%	SA: 1–8,10,11; US: n/a; NA: 9 (College selection, analysis intentions & classification of some variables unclear)
**Medical/ dental practitioners**	56 (2011)	2006; Ernakulam City (86% U)	n = 114, dentists, 54.4% male, ages NR	17.6% current smokers, 13.2% ex-smokers	Self-administered questionnaire	**Use of tobacco histories**: 60.9% use with 50% of patients, 10.6% never use; 54.6% **not confident in tobacco cessation**, no provision of **assistance with quitting or referral**	SA: 1–3; US: 7,10,11; NA: 4–6,8,9 (Intended analysis unclear, selective reporting possible)
**Medical/ dental practitioners**	30 (2004)	2004; Bihar (U/R NR)	n = 521 doctors; 55.3% GPs; 82% 25–55 years; 89.3% male	Cigarette smokers: 7%; ‘other tobacco users’: < 1%; `chewing/ applied product’ users: 11.7%	Self-administered questionnaire	**Use of tobacco histories:** 40.4% in adults, 16.9% in paediatrics; **advise quitting:** 68.6%, & **cutting down:** 48.3%; **relate patient’s problems to tobacco**: 60.4%; **discuss benefits of quitting**: 69.2%	SA: 1,3,4,8,11; US: 2, 9, 10; NA: 5–7 (Aims unclear, data collected & analysis intentions NR, some reported outcomes ambiguous)
**Medical/ dental practitioners**	50 (2009)	2003; Kerala (U & semi-U)	n = 110 male faculty, 15 male & 75 female doctors; mean age 42.2±7.7 years; 77.9% male	63.1% never smokers	Self-administered questionnaire	**Use of smoking histories**: 41.2% occasionally, 22.3% almost always, 35.3% always; **advise quitting**: 21.2% occasionally, 77% almost always; **assist with drugs for quitting**: 10%; **received training in cessation**: 31.5%	SA: 1–4, 8; US: 7, 9–11; NA: 5, 6 (Extent of data collection & analysis unclear, low participation rates in some groups)
**Medical/ dental practitioners**	51 (1995)	Dates NR; Punjab (U and ‘semi-U’)	n = 106; private GPs; age NR; 90.6% male	7.5% smokers	Self-administered questionnaire	**Use of smoking histories:** 22.6% always, 52.8% often, 20.8% rarely; **advise against smoking:** 18.9% regularly, 81.1% when acute presentation; no referrals made to specialist clinics; **knowledge updated via**: newspapers: 73.4%, television: 66%, journals: 3.8%, conferences/education programmes: 0	SA: 1–4, 8; US: 9–11; NA: 5–7 (Data collection methods & data collected NR, no analysis reported)
**Medical/ dental practitioners**	52 (1991)	Dates NR; Mumbai (U)	n = 363; GPs; age NR; 85.4% male	Smokers: 7.7%; smokeless users: 8.7%	Self-administered questionnaire	**Routinely advise to quit smoking**: 64%; **advise to quit smoking only if symptoms present**: 36%	SA: 1–4, 8; US: 7, 9–11; NA: 5,6 (Data collection methods, data collected & intended analysis unclear)
**Patients**	57 (2012)	2009–10; all India (31% U)	n = 25175; > 21 years; 69.6% male	100% ever tobacco users	Secondary analysis of GATS data	Extent of **use of cessation aids** approx. 10% (6.82% counselling, 1.16% alternative therapy, 0.74% NRT, 0.72% prescription medication, 0.56% ‘quitline’)	SA: 1–3, 5–11; US: n/a; NA: 4
**Patients**	37 (2009)	2006–07; Kerala (U)	n = 100; patients with diabetes; mean age 55.8±11.9 years; 100% male	100% smokers	Interview	**Asked about tobacco use at diagnosis**: 75%; **advised to quit**: 52%; **advised to cut down**: 21%; **not asked about tobacco use in past year**: 42%; **asked about tobacco use only once**: 22%	SA: 1,2,4,8,11; US: 10; NA: 3, 5–7, 9 (Interview methods, interview content & intended analyses unclear)
**Patients**	58 (2008)	2006–07; Kerala (U/R NR)	n = 215; completed TB treatment; mean age 49.0±12.1 years; 100% male	94.4% ever users; 20.2% quit 6 months prior to TB diagnosis	Interview	**Asked about tobacco use by health staff:** 99.5%; **received TB-specific smoking advice:** 49%; given **general advice to quit without explanation**: 50%; most received one brief message about tobacco at time of diagnosis	SA: 1–10; US: 11; NA: n/a (Recall bias possible)
**Medical/ dental trainees**	48 (2013)	2008–10; Kerala & Karnataka (U/R NR)	Medical faculty/students: n = 713/2585; mean age 32.9±9.7/20.3±1.7 years, 59%/47.7% male	28% faculty, 26% students ever smokers	Self-administered questionnaire	No **cessation training** received	SA: 1–8, 10, 11; US: n/a; NA: 9 (College selection, classification of some variables & analysis intentions unclear)
**Medical/ dental trainees**	26 (2013)	Dates NR; Karnataka (U/R NR)	n = 456, final year dental students, 30.5% male, mean age 22.7±0.94 years	9.1% current users, 1.3% former users	Self-administered questionnaire	**Routine use of tobacco histories**: 94.8%; **received teaching on anti-tobacco advice**: 81.9%; **give counselling**: 94%; **consider own counselling skills insufficient**: 49.8%; **never suggest use of NRT**: 76.5%; **cessation information displayed in institution**: 87%	SA: 1–6,8; US: 10,11; NA: 7,9 (Data collected unclear, possible selective reporting)
**Medical/ dental trainees**	27 (2011)	2009; Karnataka (U/R NR)	n = 329; dental students of 3 colleges in Karnataka; 20–26 years; 29.7% male	7% current smokers; 5% ex-smokers	Questionnaire (delivery method unclear)	**Routine use of tobacco histories**: 93%; **give cessation advice**: 94.2%; **received teaching on anti-tobacco advice**: 47.1%; **counselling considered ineffective unless patient’s problem related**: 67.8%; **cessation information displayed in institution**: 58.7%	SA: 1, 2, 4–7, 9; US: 8, 10, 11; NA: 3 (Convenience sample, multiple comparisons)
**Medical/ dental trainees**	28 (2012)	2005 & 2009; all India (U+R)	Medical/dental students: n = 1176 (2005), 1523 (2009)/ 1339 (2005), 711 (2009); age, sex NR	Medical/dental students: smokers: 13.4%/6.5%; smokeless users: 11.6%/8.6%	Secondary analysis of GHPSS data	No S change in **provision of cessation training** for medical students between 2005 & 2009, but S increase in training received by dental students (mean 10.5 to 54.8%)	SA: 1, 3–9, 11; US: 10; NA: 2
**Medical/ dental trainees**	49 (2011)	2007; Bangalore (U)	n = 76; clinical residents; mean age 28±2.9 years; 68% male	NR	Self-administered questionnaire	> 80% **routinely use tobacco histories**; 50% **routinely offer cessation advice;** 2/3 **wait for patients to request assistance**; self-rated as low/average **proficiency in cessation practice**: 69%	SA: 1–4, 7–10, 11; US: n/a; NA: 5, 6 (Small n-number, questionnaire components NR)

Reviewed studies relating to FCTC Article 14 included in all aspects of synthesis, sub-categorised by population surveyed. The numbers following the different quality categories (SA, US, NA) indicate the aspect of quality assessment (see Table 2), rated as satisfactory (SA), unsatisfactory (US) or not-assessable (NA). All studies were of cross-sectional design, or secondary analyses of cross-sectional surveys. U = urban; R = rural; GP = general practitioner; NR = not reported; GATS: Global Adult Tobacco Survey; TB = tuberculosis; NRT = nicotine replacement therapy; GHPSS: Global Health Professions Student Survey; S = significant; NS = not significant

**Table 7:** Studies related to trialled interventions.

Relevant FCTC Article and intervention type	Ref. (pub. year)	Study dates, design & location	Sample size & characteristics	Intervention	Methods (M) & follow-up (F)	Main outcome measures (bold) & results	Quality assessment (numerical ratings and main concerns)
**12: Education, communication, training and public awareness (school-based interventions)**	59 (2012)	2010–2011; QE; Maharashtra (U/R NR)	Controls: n = 690 8^th^ graders, mean age: 13.6 years, 51% male; 8.7% tobacco users; Intervention group: 8^th^/9^th^ graders: n = 660/501; mean age: 13.4/14.5 years; 48%/46% male; 4.1%/3.6% tobacco users	Year 1: education programme; Year 2: work with civic authorities/other organisations	M: Follow-up by survey; F: at 1 year (8^th^ grade intervention group) or 2 years (9^th^ graders) post-start of intervention, unclear for controls	Control/intervention group: **awareness of programme**: 16%/97–99%; **read programme newsletter**: 5%/40–50%. S higher **knowledge**, **life skills, self-efficacy scores,** and **actions to prevent tobacco use among others**, in intervention group. S less **tobacco use** in intervention group.	SA: 1–2,7,10–11; US: n/a; NA: 3–6, 8,9 (No pre-intervention data, one control group for heterogeneous intervention groups, analysis methods/ outcomes unclear)
**12 (school-based interventions)**	60 (2013)	2004–06; RCT cost analysis; Delhi & Chennai (U)	n = 6365; school students; 10–16 years; 43% female	Project MYTRI: classroom curriculum, posters, parent postcards & peer-led health activism	M: theoretical cost analysis; F: lifelong modelling study	4.52 **QALYs added per averted smoker**; **cost per QALY added** due to averted smoking = $2769; **cost per life-year added**: $4348	SA: 1–5, 7, 8; US: 10,11; NA: 6, 9 (Source & breakdown of costs not presented, some assumptions based on data from outwith India)
**12 (school-based interventions)**	61 (2011)	2004–06; RCT mediation analysis; Delhi & Chennai (U)	n = 6381 (intervention group), 7704 (control group); 43.4% female, ages NR (6^th^-8^th^ graders)	Project MYTRI (as above)	M: mediation analysis based on questionnaire data; F: at baseline and post first and second years of intervention	(1) **Positively affected by intervention & positive effect on behaviour**: knowledge of health effects, reasons to use/not to use, advocacy skills, normative beliefs; (2) **Negatively affected by intervention**: perceived prevalence of tobacco use	SA: 1–9; US: 10, 11; NA: n/a (Secondary analysis, size/relevance of effects unclear)
**12 (school-based interventions)**	62 (2009)	2004–2006; RCT; Delhi & Chennai (U)	n = 6365 (intervention group), 7698 (control group); school students; 43% female; ages NR (6^th^-8^th^ graders)	Project MYTRI (as above)	M: delivered over 4 months of each of 2 years; data collected via self-administered questionnaire; F: at baseline and post first and second years of intervention	**Tobacco use:** increased by 68% in controls, decreased by 17% in intervention group: S differences in **changes in cigarette** (p < 0.05), **bidi** (p < 0.01) & **any tobacco use** (p < 0.04), & **tobacco use intentions** (p < 0.03)	SA: 1–10; US: 11; NA: n/a
**12 (school-based interventions)**	63 (2010)	2004–05; process evaluation of RCT; Delhi & Chennai (U)	n = 5564; school students; sex NR, ages NR (6^th^-8^th^ graders)	Project MYTRI (as above)	M: delivered over 4 months; data collected by ‘co-ordinators’, teachers, & peers; structured data collection process; F: assessed throughout implementation of project	**Average extent to which programme implemented:** 71.3%, more **trained teachers** correlated with higher implementation rates & lower susceptibility to tobacco use; teachers ‘often’ **reported activities 'enjoyed' when 'not conducted'**	SA: 1–5, 7, 8, 11; US: 10; NA: 6, 9 (No controls, risk of recall bias)
**12 (school-based interventions)**	64 (2009)	2004–2005; mediation analysis of RCT; Delhi, Chennai (U)	n = 4360 (control group), 4009 (intervention group); school students; intention to use tobacco; 51.6% male; ages NR (6^th^-8^th^ graders)	Project MYTRI (as above)	M: mediation analysis based on survey data; F: 1 year post-start of intervention	**Intention to use tobacco used as outcome variable for mediation analysis**; S **programme effect** for intention to chew (p = 0.04) but not smoke (p = 0.07); knowledge of health effects (10% of total effect), normative beliefs (18%), reasons for use (6%) &perceived prevalence (7%) S **mediators between intervention & use intentions**	SA: 1–4,6,7,9; US: 10, 11; NA: 5, 8
**12 (school-based interventions)**	65 (2002)	1997–1999; group randomised trial; Delhi (U)	n = 1293–1863 (variation by group & pre/post-test); school students; age 12 years; 50.5% males	School based: cardiovascular health education programme; Family-based: booklets to share with families	M: Schools assigned to school/ family programme, school alone, or control condition; F: students surveyed at baseline & immediately following intervention (1 year duration)	At post-test: NS difference in ‘**tobacco knowledge’** between groups; S lower ‘**tobacco knowledge/attitude score’** among ‘school only’ cf. other conditions, S more in control cf. other conditions **tried smoking**, S more in control cf. school/family group **tobacco use intentions**	SA:1,4–6,10; US: 2,7,11; NA: 3,8–9 (Limited demographic information, some outcomes NR, limited follow-up, p-values NR)
**12 (community-based intervention)**	66 (2010)	2006–2007; QE; Delhi (U)	Controls: n = 1152 (baseline), 1083 (endline); intervention group: n = 1229 (baseline), 1162 (endline); residents of slum/resettlement colonies; 10–19 years; sex distribution NR for full population	Community-based intervention including posters, films, lectures, plays, booklets, pamphlets, awareness rally	M: 3–7 sessions of 4 activities conducted at different locations (mean 40–50 participants at each); F: questionnaire administered at baseline & 1 year post-start of intervention	At post-test: Prevalence of current (p = 0.003) & ever (p = 0.009) **tobacco use** lower in intervention cf. control group; **fresh uptake** higher in control cf. intervention groups (OR = 5.96 (95% CI: 1.73–20.51)); NS between-group difference in **quit rate**	SA: 1–2,4,6,10; US: 7, 11; NA: 3, 5, 8–9 (Area selection and sorting to groups unclear, small n-number for females, no information regarding fidelity of intervention, some outcomes NR)
**14: Demand reduction measures concerning tobacco dependence and cessation (pharmacological intervention)**	67 (2010)	Dates NR; RCT; Delhi (U)	n = 30 (15/group); smokers attending cessation clinic Drug group/placebo group: mean age 46.9±14.1 years/39.3±12.2 years, 100% male/93.3% male	Drug group: physician advice + bupropion; Placebo group: advice + placebo	M: Single-blind RCT; abstinence, weight, Beck’s depression score, withdrawal symptoms & side effects monitored; F: intervention for 7 weeks, follow-up at weeks 1–8, 12 & 16	**7-day abstinence rate (drug vs control):** week 2: 46.67 vs 13.33% (p = 0.04); week 16: 53.33 vs 20% (p = 0.05); NS difference in **depression scores**; mean **weight gain** S less in drug cf. placebo group, **withdrawal symptoms** S higher in drug group in early weeks. **Side effects** higher in drug group	SA: 1,3,5–6,9,11; US: 2,7,10; NA: 4,8 (Small n-numbers, limited follow-up, recruitment & randomisation methods unclear)
**14 (non-pharmacological interventions)**	68 (2013)	2008–2011; RCT; Kerala (‘peri-U’)	n = 196 (98/group); smokers with diabetes; > 18 years, mean ages 54.2 years (control group), 52.5 years (intervention group); 100% male	Both groups: advised to quit, diabetes education materials; Intervention group: 3 diabetes-specific counselling sessions	M: parallel group pilot RCT; counselling sessions run over 3 months; F: at 1, 3 and 6 months post-start of intervention	Control group/intervention group at 6 months: **quit rate**: 12.5%/51.8% (p < 0.001), **harm reduction**: 25.5%/37.0% (p = 0.101)	SA: 1–8, 10, 11; US: n/a; NA: 9 (Analysis intentions unclear, possible misclassification as 7 day abstinence = ‘quit’; limited follow-up)
**14 (non-pharmacological interventions)**	69 (2012)	Dates unclear; RCT; Tamil Nadu (R)	n = 181 (intervention group), 185 (control group); tobacco users; 20–40 years; 100% male	Intervention group: 2 tobacco education sessions; cessation self-help material; Control group: self-help material alone	M: cluster randomised trial; 5 weeks between education sessions; F: interview at baseline & 3 weeks post-end (2 months from start) of intervention	Intervention/control group: **abstinence**: 13.8%/6.5% (p = 0.016); **quit attempt**: 30.1%/21.4% (p = 0.033); **harm reduction**: 24.4%/9.8% (p = 0.003)	SA:1–2,4,5–7,9–11; US: 3,8; NA:n/a (12.5% participation from randomly selected sample, not blinded, limited follow-up)

Reviewed studies of trialled interventions, by FCTC Article. The numbers following the different quality categories (SA, US, NA) indicate the aspect of quality assessment (see Table 2), rated as satisfactory (SA), unsatisfactory (US) or not-assessable (NA). NR = not reported; RCT = randomised controlled trial; U = urban; R = rural; NS = non-significant; S = significant; QALY = quality-added life year; QE = quasi-experimental study; OR = odds ratio; CI = confidence interval

### Studies describing the extent of tobacco control measures

#### Article 6: Price and tax measures to reduce the demand for tobacco (Table 4)

The one identified study in this category suggested a 2011 increase in taxation had led to a reduction in self-reported tobacco sales and consumption at the short-term end-point (less than one month post-intervention) considered [25].

#### Article 8: Protection from exposure to tobacco smoke (Table 4)

The five studies in this category included in full analysis reported on prohibition of smoking on clinical campuses and SHS exposure. Data collection relied on self-reported outcomes. Four studies reported on smoking policy. In a 2004 study, 49.1% of doctors reported no local smoke-free policy [30]. Higher proportions of students (> 85%) reported smoke-free policies on campus in two later studies, both likely to have taken place post-implementation of FCTC [26,27]. However, in a secondary analysis of GHPSS data, no trend towards a wider extent of smoke-free policies (between 2005 and 2009) was observed (40.8% in 2006 and 2009 reported for medical schools; for dental schools: 67.6% in 2009, 72.6% in 2006) [15,28]. According to the GSPS, 68.7 and 65.2% of school personnel reported school policies against student and staff tobacco use, respectively, in 2009 (66.9 and 57.1% in 2006) [15].

Two secondary analyses of GTSS data reported on recent trends in SHS exposure among school students and medical/dental students. One suggested SHS exposure had declined among dental, but not medical, students between 2005 and 2009 [28]. GHPSS data indicate that most medical and dental students are nevertheless still exposed to SHS, particularly in public places (52.5% of dental students and 71.4% of medical students exposed in 2009) [15]. Exposure among nursing students (50.8% in 2007) and pharmacy students (55.0% in 2008), was similar. The second study suggested that SHS exposure declined among young people between 2003 and 2006 (from 36.4% to 26.6% at home, 48.7% to 40.3% in public places) [29]. Updated GYTS data suggest this downward trend continues, but that SHS exposure among young people is still widespread and more common in public places than at home [15]. In contrast, among adults, exposure at home (40%) is more common than exposure at work (29.9%) and in public places (5.4–17.5%; 2009 GATS) [15].

#### Article 11: Packaging and labelling of tobacco products (Table 4)

The GATS data (2009) indicate that 54.7% and 62.9% are aware of health warnings on cigarette and smokeless tobacco packaging, respectively [15]. One additional identified study (published 2011) demonstrated that although 25.6% of young people from tribal villages were aware of warnings on tobacco packaging, fewer (21.0% of those aware) could interpret these as intended [31].

#### Article 12: Education, communication, training and public awareness (Table 5)

Of the studies in this category included in all aspects of analysis, five related to knowledge and education among young people, eleven to adult knowledge/education, and ten to knowledge and training of healthcare professionals. All relied on self-reported outcomes. Outcomes investigated included knowledge of the consequences of tobacco use and SHS exposure, beliefs about medicinal uses of tobacco, and inclusion of tobacco in school and health professional training curricula.

Of the identified studies relating to young people, three were direct investigations of tobacco-related knowledge. A 2000 survey suggested that 29.1%, 22.4% and 41.3% of tobacco-using schoolchildren were aware of the harms associated with smoking, smokeless tobacco and SHS exposure, respectively [35]. Corresponding prevalences among non-users were 62.9%, 62.1% and 59.2% [35]. In a more recent study (published 2011), 94.2% of ‘tribal adolescents’ were aware that tobacco is harmful [31]. A 2008 study suggested knowledge of specific risks was less common and varied from < 1% (hypertension and heart disease) to 61.8% (cancer). 10% were aware of tobacco’s addictive properties [32]. In the same study, 72% of females and 25.6% of males reported use of snuff for tooth cleaning and 97% accepted tobacco as a medication for abdominal pain or toothache.

Regarding school-based education, two secondary analyses of pre-FCTC GTSS data were identified. One demonstrated high variability in the teaching of knowledge/skills to prevent youth tobacco use, between two states [33]. The second suggested that students in federal schools recalled tobacco-related teaching more than those in state schools [34]. In the most recent GSPS (2009), 44.4% of staff reported the inclusion of tobacco use prevention in their school curriculum (cf. 42.0% in 2006), 10.1% of teachers had received relevant training (cf. 16.7% in 2006), and 37.8% had access to relevant materials (cf. 34.6% in 2006) [15]. In line with these outcomes, 2009 GYTS data suggest 63.3% of students receive teaching about tobacco-related harms (cf. 54.4% in 2006) [15].

Regarding adult education, a study of a 2009–10 Government television/radio campaign showed that in the weeks after airing it was recalled by 65% of tobacco users, who generally considered it relevant [36]. This compares to the 36% and 15.1% reported to have recalled anti-cigarette messages from television and radio, respectively, in the 2009 GATS [15]. This additionally reported on viewing of anti-cigarette information in newspapers/magazines (25.1%) and on billboards (21.5%). One further related study (published 2011) suggested 69% of ‘tribal adolescents’ had heard radio/television tobacco prevention messages, but only 1.8% of those exposed could interpret the messages as intended [31].

Nine studies of adult knowledge outcomes were identified. Four were studies of school personnel (all published 2004). In these, the proportion of participants that considered tobacco harmful (66.4%, 78.4%, 88.6%, 88.9% and 94.2%), to be addictive (84.5%, 88.6%, 90.7%) and to be harmful via SHS exposure (88.5%, 90.8%, 92.3%), were reasonably high [42–45]. Many others (79.4% of a smoking population, 82.8% and 97.3% of general populations, and 71.6% and 64% of smokeless tobacco-using populations)—all likely to have been surveyed post-FCTC—also considered tobacco use harmful [38–41]. However, fewer considered it harmful to themselves specifically, and some considered particular levels of smoking safe [37–39]. As among young people, there was also a lower appreciation of specific health effects, and an indication that tobacco use was considered to have some positive effects [37,40,41]. The 2009 GATS data are generally consistent with these outcomes (90.2%, 88.8% and 82.9% reported smoking, smokeless tobacco use and SHS exposure to be harmful, respectively), and as in all other studies reviewed, the link between tobacco use and cancer was more commonly appreciated than links with other diseases (e.g. 84.9% appreciated the link with lung cancer, compared with 49.4% a link with stroke) [15].

The ten studies of knowledge outcomes among health professionals included seven surveys of doctors, three of dental students, one of medical students, and one medical curriculum review. In an early study (published 1991), 97% of doctors considered tobacco use harmful [52]. 90.4% suggested no level of tobacco use was safe in a 2004 study [30], but other studies (2003, 2007 and 2009–10) suggested that only one quarter of medical faculty consider smoking more than six times per day harmful, that one quarter of residents consider tobacco use a ‘fun activity, not to interfere with’, and that 11% of faculty and 46% of students consider various levels of tobacco use safe [48–50]. As among non-health professionals, despite generally widespread appreciation that tobacco causes harm, links with specific diseases were less well appreciated [49], and evidence from one study suggested that cancer was again the disease most commonly associated with tobacco use [46]. In two recently published studies (2011 and 2013), more than 97% of dental students reported having been taught about the link with cancer [26,27]. In contrast, a 2009–10 curriculum review in five medical colleges suggested tobacco-related teaching was not systematic or sufficient. 64% of students at these colleges reported receipt of some tobacco-related teaching, and generally it was felt to be ‘minimal’ [48]. The 2009 GHPSS indicates 86.6% of dental students received teaching about tobacco-related harm (cf. 97.2% in 2005). 73.5% of medical students reported such teaching in 2009 (cf. 80.7% in 2005) [15].

Regarding cessation, knowledge of options for input was reported to be much higher in a 2011 study [47] than in an earlier study (published 1995) [51], but still only 17% of participants felt sufficiently knowledgeable and skilled to deliver cessation services. 20% felt sufficiently prepared in a 2009–10 study [48]. Further information about cessation-related training is discussed in relation to Article 14.

#### Article 13: Tobacco advertising, promotion and sponsorship (Table 4)

The four studies related to Article 13 included in all aspects of analysis utilised school student survey data. Results from 2000 and 2004 suggested that almost all school students recalled exposure to advertisements [55], although fewer (238/11624) could recall brand names [54]. A secondary analysis of GYTS data indicated that 37.8% of schoolchildren were exposed to billboard advertisements in 2006, similar to levels in 2003 [29]. In 2009, 7.3% of students surveyed reported owning a tobacco-branded item, and on average they had viewed 60% of a list of movies depicting tobacco use [53]. In the GYTS survey, 8.1% of young people reported having been offered free cigarettes by a tobacco company in 2009 (cf. 9.3% in 2006) [15]. 2009 GATS data suggest that relatively fewer adults are aware of tobacco advertising: frequency of recall did not exceed 10.7% for any advertising method, and awareness of tobacco promotions was low among adults, not exceeding 4% for any type of promotion [15].

#### Article 14: Demand reduction measures concerning tobacco dependence and cessation (Table 6)

The identified studies in this category included surveys of healthcare professionals, healthcare students, and patients. Outcomes considered related to training and practice in tobacco control. In two pre-FCTC surveys of doctors (2003 and 2004), and a post-FCTC survey of dentists (2006), approximately half reported routine use of tobacco histories [30,50,56]. Pre-FCTC, most doctors (64%, 68.6% and 77.5%, in different surveys) reported routine provision of cessation advice [30,50,52]. In contrast, two post-FCTC (2006–07) surveys of patients with diabetes and tuberculosis suggested most had been asked about tobacco use, but only approximately half advised to quit [37,58]. In the 2009 GATS, 53.0% had been asked about smoking, 34.2% about smokeless use, and 46.3% of smokers and 26.7% of smokeless users recalled being advised to quit [15].

Regarding cessation assistance, post-FCTC studies suggest that 54.6% of dentists (in 2006), and 80–83% of doctors (2009–11), feel they have insufficient experience to offer cessation assistance [47,48,56]. Only small numbers report offering assistance: 10% of doctors in one study [50], none of the dentists surveyed in another [56]. In the 2009 GATS, 9.2% and 7.6% of smokers and smokeless tobacco users had received cessation counselling/advice, and 4.0% of smokers had used pharmacotherapy as a cessation aid [15]. A recent analysis of GATS data suggested alternative (e.g. ayurvedic) methods are used more commonly than NRT [57]. 31.5% of doctors had received cessation-related training in a 2003 survey [50], and 16.5% work-based training in a 2011 survey [47].

A secondary analysis of GHPSS data reported a significant increase in cessation training for dental students between 2005 and 2009 (54.8% prevalence in 2009, cf. 10.5% in 2005), but only a small upward trend among medical students (29.1% in 2009, cf. 22.3% in 2005) [28]. In keeping, more recent surveys reported receipt of cessation–related teaching by 47.1% and 81.9% of dental students [26,27], but little training for medical students [48]. 35.1% of nursing students had received cessation training in 2007, and 30.4% of pharmacy students in 2008 (GHPSS) [15].

All available studies of tobacco-related practice among medical and dental trainees are post-FCTC. They suggest the majority (> 80%) make routine use of tobacco histories. Most dental students gave cessation advice [26,27]. Despite this, and the high levels of training reportedly received by students in one study, 49.8% nevertheless still considered their counselling skills insufficient, and only 23.5% had suggested use of NRT to those intending to quit [26]. Similarly, 69% of medical residents self-reported low/average abilities in cessation assistance [49]. 50% did offer cessation advice, but most waited for patients to request it.

#### Article 16: Sales to and by minors (Table 4)

A 2000 study suggested that most 13–15 year olds could buy tobacco without difficulty [55], and a secondary analysis of GYTS data indicated that the proportion of young users purchasing tobacco in stores was unchanged between 2003 and 2006, and that the number of young people offered free cigarettes from tobacco companies significantly increased over the same period [29]. The updated GYTS data (2009) suggest that 47% of young people usually buy their cigarettes in a store (cf. 51.9% in 2006), and 56.2% were not refused purchase due to age (cf. 72.1% in 2006) [15].

### Studies relating to trialled interventions (Table 7)

Of the 25 identified studies relating to trialled interventions, eleven were without reliability concerns. Eight related to FCTC Article 12 (Education, communication, training and public awareness), and three to Article 14 (Demand reduction measures concerning tobacco dependence and cessation). Only one study was carried out prior to the FCTC.

#### Interventions relating to Article 12: Education, communication, training and public awareness

The studies relating to Article 12 included seven of school-based interventions, and one of a community-based intervention. Five of the school-based interventions related to Project MYTRI (Mobilizing Youth for Tobacco-Related Initiatives in India; details in Table 7), and aimed to determine the extent of implementation, impact on tobacco use, associated knowledge, life-years added, and mediators of its effect. The other school-based projects had similar aims/methods and measured tobacco use and knowledge outcomes.

Results from the Project MYTRI studies suggested it was implemented at just over 70% of intended levels, and impacted positively on tobacco use, and (via projection) life years [60,62,63]. Knowledge scores, advocacy skills, ‘normative beliefs’ and teacher training were found to be potential mediators of effect [61,63,64]. One of the two other tested interventions was also associated with higher knowledge scores, and advocacy skills, and less tobacco use [59]. In the second study, effect on tobacco-related knowledge was less convincing [65]. One possible reason for this is that this study aimed to address all behaviours linked to cardiovascular health, rather than smoking alone. The eight study in this category was a multicomponent community-based intervention that appeared to have a positive impact on tobacco use in slum/resettlement areas [66].

#### Interventions relating to Article 14: Demand reduction measures concerning tobacco dependence and cessation

One of the studies reviewed under this Article was a pharmacological intervention that investigated the effect of bupropion as an adjunct to cessation counselling. It had a low n-number and short follow-up period, but was suggestive of more favourable outcomes in those receiving treatment [67].

**Fig 2:**
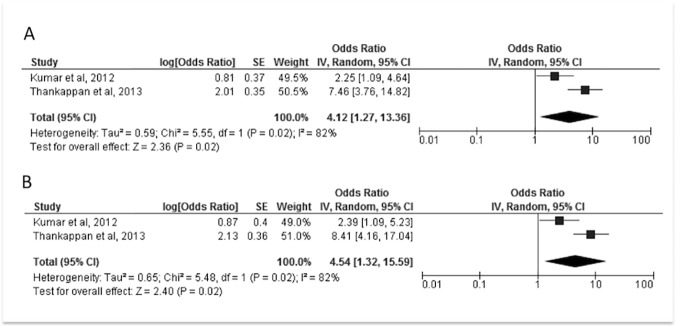
Meta-analyses of data from non-pharmacological cessation interventions. Forest plots, effect estimates and measures of heterogeneity, relating to the meta-analyses performed with unadjusted (A) and adjusted (B) data are displayed

The two additional studies reviewed investigated the impact of counselling or education sessions as an adjunct to self-help materials. One intervention was delivered generally to tobacco users [69], the second to smokers with diabetes [68]. Both appeared to be of positive impact. The results of the adjusted and unadjusted meta-analyses relating to quit rates are shown in Fig 2. The odds ratio for quitting in intervention versus control condition was 4.12 (95% confidence interval: 1.27, 13.36) using unadjusted data, 4.54 (1.32, 15.59) using adjusted data. Chi-square tests demonstrated heterogeneity (p = 0.020 for both analyses), and I^2^ values were 82% for both analyses. This is likely to be associated with the different characteristics of the study populations, the different follow-up durations, and the use of cluster randomisation in one study, that we were unable to adjust for with the information available. In view of this heterogeneity, the small number of studies, the short-term follow-up, differential adjustment of results between studies, and definition of cessation used (seven-day abstinence), we are cautious about the magnitude of the impact of the interventions, in both the pooled and un-pooled results. Such interventions nevertheless appear of benefit.

### Subgroup analysis

Although subgroup comparisons were made within some of the studies reviewed, these are not reported as they were so few and diverse that between study comparisons was not feasible. However, some studies indicate that major subgroup differences may exist. For example, knowledge of tobacco-related harm is reportedly lower in tobacco-using, compared with non-using, children [32]. State, urban/rural status, sex and education have been described as predictors of tobacco-related knowledge [38], and variation in tobacco-related teaching by school type and location has been observed [33,34]. Data also suggest that tobacco marketing is effectively targeted towards younger people, and hint that anti-tobacco messages are not understood by all sub-populations [31]. Subgroup differences will not be applicable to all types of intervention, but will be useful to consider in application of interventions, to avoid discrimination, and because those that impact on different groups are likely to produce synergistic effect. Further investigation of such putative associations will therefore be of interest, once sufficient comparable data exist.

## Discussion

Additional to the GTSS data, eighty studies were identified for review. There was a high degree of variability in study design, location, population characteristics, and outcomes, and most studies relied on self-reported outcomes. There were concerns regarding the reliability of results in many cases—including among recent studies. In some cases this was explained by the particular intentions of the investigators, but nevertheless 35 studies (and parts of four further studies) were dropped from our analysis of outcomes. This left 45 studies for inclusion: 34 describing various aspects of tobacco control, 11 the results of trialled interventions. The majority of identified studies related to FCTC Articles 12 and 14.

Related to Article 12, awareness that tobacco use and SHS exposure is harmful, was reasonably high among all populations investigated (> 60% in all cases). Knowledge of more specific consequences of tobacco use was less frequent. Personalisation of the potential harm was also relatively uncommon, and there was evidence of beliefs in some medicinal uses of tobacco [e.g. 35,41]. There was no evidence that medical professionals considered tobacco to have such uses, but a substantial proportion considered lower levels of tobacco use of limited consequence [e.g. 48,49]. Some were aware of cessation aids, but few felt sufficiently well equipped to use them [e.g.^47^,^48^]. For most outcomes study numbers were insufficient to identify trends associated with implementation of the FCTC, but overall knowledge of tobacco-related harm may have improved among adults post-FCTC. There was no clear indication of similar positive trends among health professionals.

For schoolchildren, teaching about tobacco use appears to have recently become slightly more extensive, but marked variation between regions/schools remains [33], and the latest GSPS suggested little training or resources for teachers [15]. In keeping, trials of school-based education interventions demonstrated a positive impact on knowledge, advocacy skills and tobacco use [59,61,62,64]. Teaching about the risks of tobacco use for health professional trainees appeared more widespread, but may have reduced slightly post-FCTC. Community-based education interventions and education interventions for adult tobacco users appeared beneficial [66,69].

Related to FCTC Article 14, doctors and patients reported providing and receiving only low levels of cessation assistance, respectively [15,50,56,57]. Advice to quit was reportedly given in the majority of cases of identified tobacco use [30,50,52], but likely remains inadequate as less than half of those surveyed reported routine use of tobacco histories [30,50,56]. There was evidence that cessation-related training for medical trainees remains insufficient, but that—whilst still far from universal—it has improved for dental students post-FCTC [28]. Although to date of limited follow-up, RCTs of education, cessation counselling and cessation assistance interventions for adults indicate that they are beneficial [67–69].

In keeping with the observed scope for enhanced education/cessation interventions—as called for by the FCTC, and as trials suggest could be successful in India—the Government of India has recently disseminated guidelines for tobacco education to schools across the country [19]. Data regarding the extent of implementation and intended effects are awaited. In contrast, although they do also form part of the National Tobacco Control Plan, there is as yet no evidence for enhancement of tobacco-related education and prevention/cessation training in medical and dental school curricula, or the production of training programmes aimed at current medical/dental practitioners. The latest disseminated information suggests the Government of India plan to implement specialist cessation services as distinct units—separate from traditional health service provision, and run by specially trained professionals [70]. This has also been suggested for the dental profession [71], and there is evidence that small numbers of such services do exist [71,72]. How much further these plans have been enacted is unclear and we did not identify any studies relating to such services and their outcomes. We are therefore unaware of the extent to which they are available and used, and any associated outcomes. Regardless of their existence, supplementary education about tobacco would still be beneficial to traditional healthcare providers, and indeed may be necessary if such novel initiatives are to be successful.

The reviewed studies relating to FCTC Article 8 were indicative that overall, smoke-free policies are becoming more widespread post-FCTC, with concomitant downward trends in SHS exposure. Nevertheless, SHS exposure remains high among all groups, including children. Regarding Article 13, pre-FCTC studies suggested most young people were exposed to tobacco advertisements. One study suggested no downward trend immediately post-FCTC, but more recent data are lacking. Another study suggested more adolescents were offered free cigarettes by tobacco companies post-legislative changes [29], in keeping with evidence from India and elsewhere indicative that prohibition of tobacco advertising has led to tobacco companies forming contractual agreements regarding brand display with the film industry [73,74], and evidence that images of tobacco use in Bollywood movies increased post-implementation of the COPTA [73,75]. The reviewed studies relating to Article 16 indicate that it remains easy for minors to purchase tobacco [15,29]. Adults appear to be less exposed to tobacco promotions.

One study related to FCTC Article 6 was identified for review. This suggested that a taxation policy had positive impact on tobacco consumption [25]. Taxation strategies are an important issue for India. A recent modelling study suggested they could be particularly meaningful [76], and India has consistently scored relatively poorly in related indices [4,8,9]. Although tobacco companies have had some influence on this, associated problems relatively specific to India include the unregulated tobacco industry, welfare of small manufacturers and need for alternative crop/employment strategies. Data relating to taxation strategies and/or these related issues are lacking. Nevertheless, the newly instated Indian Government has just increased taxes on cigarettes. The opportunity to assess impact on these outcomes therefore now exists. It is also possible that recent intentions to establish tobacco testing laboratories, implement alternative crop programmes and re-train tobacco workers have moved forward [11], but we did not identify any data relating to these strategies or otherwise related to FCTC Articles 9 and 17. Data regarding Article 11 were limited, and no studies of the remaining FCTC Articles relating to demand- or supply- reduction measures were identified (Articles 7, 10 and 15).

### Limitations of review

Although an important outcome in itself, the number and diversity of studies available for review limited the scope for synthesis, including meta-analysis of trial data. As exclusions were made where insufficient information was available for adequate assessment of potential methodological concerns, as well as for specific concerns, some potentially useful data may have been omitted, although we note that the outcomes of such excluded studies were never at particular odds with our main outcomes.

Among the included studies, concern about the relevance of some results persists. For example, in the trials reviewed, follow-up was frequently short, which is potentially important as there is a lack of data regarding relapse rates in smoking cessation and the long-term benefits of interventions (e.g. [77,78]). Additional limitations that impact on our ability to draw rigorous conclusions include the extent of reliance on self-reported outcomes and a lack of recent data for some outcomes. This limited review of the impact of the FCTC, including associated recent developments, such as the recent move to implement a nationwide school-based tobacco curriculum. Further limitations linked to our own methodology included the possible impact of publication bias—particularly given the largely positive outcomes of the interventions reviewed—and the possible limited generalizability of some of the included studies, given the specific population characteristics, and limited numbers, in some cases.

### Implications of review outcomes

Together with the recently stated intentions of the Indian Government, and the long-standing National Tobacco Control Plan, these results provide opportunities and suggestions for both research and policy/practice. Although the details of the new government’s plans are awaited, it has been suggested that the overarching aim will be enhanced compliance with the FCTC. Wide-ranging interventions can thus be anticipated. Not only will these address aspects of tobacco control that have been little studied in India to date, but successful in other countries; they will also aim to impact on areas of control relatively specific to India—such as facilitation of alternative crop production, and training and development options for current tobacco workers. Indeed there is some evidence of steps already taken by previous governments to move such issues forward [11].

These proposed changes would provide an opportunity to fill the data gaps that exists for several potential tobacco control strategies in India, highlighted by this review. The low number of studies available and concerns about reliability of some also indicate, however, that an enhanced capacity for research would be a useful component of any proposed strategy, and necessary if the trials are to be helpful in guiding future policy changes. As data from low- and middle- income countries is generally relatively limited, the outcomes would be of wider interest, and the first relating to some forms of intervention less relevant to higher-income countries.

In the meantime, the data available for review support the introduction of several particular interventions. Firstly, the development of a comprehensive tobacco-related curriculum for medical and dental schools, and teacher training colleges, to complement that being rolled-out for schoolchildren, is likely to be useful. A plan to educate those already in practice, and a mechanism to facilitate continuous knowledge update, is also desirable. There has been some indication of an acknowledgment that this is required—and may follow the education of those involved in higher management of tobacco control strategies—but no information about the specifics [70,71,79]. Until medical practitioners and faculty are updated on tobacco-related subjects, relatively specific and comprehensive guidelines may be required. Cessation-related training appears to be a particular need, and studies have suggested this would be welcomed by these groups [e.g. 49,50].

Our review has also indicated that there may have been enhanced marketing of tobacco products towards young people over recent years. Others have reported the tobacco industry’s use of surrogate and indirect advertising methods, including use of internet-based marketing, violations of some legal provisions regarding advertising (achieved mainly through a lack of enforcement capacity), and use of legal proceedings to delay introduction of new control policies [11,74]. Together these occurrences suggest that ongoing review of industry methods, enhanced legal capacity, and an enhanced capacity to enforce related legislation, would all be useful. Indeed our results indicate that many aspects of tobacco control would benefit from enhanced policy enforcement capacity. This need is likely only to increase further as further policy is introduced. Recently, an India-based NGO has demonstrated that attempts to enhance capacity are likely to lead to positive outcomes [16,17]. Working with such stakeholders could be a positive move for policymakers.

## Conclusions

Tobacco-use outcomes could be improved by school/community-based and adult education interventions, and cessation assistance, facilitated by training for health professionals and schoolteachers. Smoke-free policies appear to have become more widespread post-FCTC, but further, more up-to-date data describing this, SHS exposure, tobacco advertising/promotions, and availability to minors, would be useful. To date, data relating to taxation/pricing and tobacco packaging appear to be few, and we did not identify any studies of product regulation, alternative employment strategies, or illicit trade. Further investigation of these additional tobacco control measures would be of use.

## Supporting Information

S1 PRISMA Checklist (DOC)

S1 File Tables A—E in S1 File. **Table A in S1 File—Studies related to FCTC articles 6, 11, 13 and 16.** Reviewed studies relating to FCTC articles 6, 11, 13 and 16 excluded from analysis of outcomes. The numbers following the different quality categories (SA, US, NA) indicate the aspect of quality assessment (see Table 2), rated as satisfactory (SA), unsatisfactory (US) or not-assessable (NA). The main reasons for concerns regarding study reliability are also listed. All studies were of cross-sectional design. * = part of study included in analysis of outcomes. U = urban; R = rural; FGD = focus group discussion; NR = not reported; COTPA = Cigarette and Other Tobacco Products Act; LSGB = local self-government bodies; S = significant; NS = not significant. **Table B in S1 File—Studies related to FCTC Article 8: Protection from exposure to tobacco smoke.** Reviewed studies relating to FCTC Article 8 excluded from analysis of outcomes. The numbers following the different quality categories (SA, US, NA) indicate the aspect of quality assessment (see Table 2), rated as satisfactory (SA), unsatisfactory (US) or not-assessable (NA). The main reasons for concerns regarding study reliability are also listed. All studies were of cross-sectional design. * = part of study included in analysis of outcomes. NR = not reported; U = urban; R = rural; LSGB = local self-government bodies; SHS = second-hand smoke; COTPA = Cigarette and Other Tobacco Products Act; FGD = focus group discussion; S: significant; NS: non-significant. **Table C in S1 File—Studies related to FCTC Article 12: Education, communication, training and public awareness.** Reviewed studies relating to FCTC Article 12 excluded from analysis of outcomes, by sub-category. The numbers following the different quality categories (SA, US, NA) indicate the aspect of quality assessment (see Table 2), rated as satisfactory (SA), unsatisfactory (US) or not-assessable (NA). The main reasons for concerns regarding study reliability are also listed. All studies were of cross-sectional design. * = parts of study included in analysis of outcomes. U = urban; R = rural; FGD = focus group discussion; NR = not reported; SES = socioeconomic status; LSGB = local self-government body; FCTC = Framework Convention on Tobacco Control; COTPA = Cigarettes and Other Tobacco Products Act; SHS = second hand smoke. **Table D in S1 File—Studies related to FCTC Article 14: Demand reduction measures concerning tobacco dependence and cessation.** Reviewed studies relating to FCTC Article 14 excluded from analysis of outcomes, by sub-category. The numbers following the different quality categories (SA, US, NA) indicate the aspect of quality assessment (see Table 2), rated as satisfactory (SA), unsatisfactory (US) or not-assessable (NA). The main reasons for concerns regarding study reliability are also listed. All studies were of cross-sectional design. NR = not reported; U = urban; R = rural; GP = general practitioner. **Table E in S1 File—Studies related to trialled interventions.** Reviewed studies of trialled interventions excluded from analysis of outcomes, by FCTC Article. The numbers following the different quality categories (SA, US, NA) indicate the aspect of quality assessment (see Table 2), rated as satisfactory (SA), unsatisfactory (US) or not-assessable (NA). The main reasons for concerns regarding study reliability are also listed. RCT = randomised controlled trial; U = urban; R = rural; QE = quasi-experimental study; CO = carbon monoxide; NS = non-significant; S = significant; NR = not reported; OR = odds ratio; CI = confidence interval; NGO = non-governmental organisation; FGD = focus group discussion
(DOCX)

## References

[1] Eriksen M, Mackay J, Ros H. The Tobacco Atlas Fouth Ed. Atlanta, GA: American Cancer Society; New York, NY: World Lung Foundation © 2012 The American Cancer Society, Inc. Available at: http://www.tobaccoatlas.org/uploads/Images/PDFs/Tobacco_Atlas_2ndPrint.pdf Accessed 2014 Aug.

[2] Jha P, Jacob B, Gajalakshmi V, Gupta PC, Dhingra N, Kumar R et al A Nationally Representative Case-Control Study of Smoking and Death in India. N Engl J Med 2008;358: 1–11. 10.1056/NEJMp0707917 18272886

[3] John RM, Sung HY, Max W. Economic Cost of Tobacco Use in India, 2004. Tob Control 2009;18: 138–43. 10.1136/tc.2008.027466 19131453 PMC2655042

[4] World Health Organization WHO report on the global tobacco epidemic, 2011: Warning about the dangers of tobacco © World Health Organisation 2011 Available at: http://www.who.int/tobacco/global_report/2011/en/. Last accessed: August 2014

[5] Goel S. India: Smokeless tobacco ban. Tob Control 2012;5: 458

[6] World Health Organisation. Global tuberculosis report 2012 © World Health Organisation 2012 Available at: http://www.who.int/tb/publications/global_report/gtbr12_main.pdf. Last accessed: August 2014

[7] Mathew JL, Patwari AK, Gupta P, Shah D, Gera T, Gogia S, et al Acute respiratory infection and pneumonia in India: a systematic review of literature for advocacy and action: UNICEF-PHFI series on newborn and child health, India. Indian Pediatr. 2011;48: 191–218. 21478555 10.1007/s13312-011-0051-8

[8] Sunley EM. India: The tax treatment of bidis Paris: International Union Against Tuberculosis and Lung Disease, 2008.

[9] ERC Statistics International Plc. The World Cigarette Market: The 2001 Survey. Suffolk, UK.

[10] Lal PG, Wilson NC, Singh RJ. Compliance surveys: an effective tool to validate smoke-free public places in four jurisdictions in India. Int J Tuberc Lung Dis 2011;15: 565–566. 10.5588/ijtld.10.0372 21396222

[11] Kaur J, Jain DC. Tobacco control policies in India: implementation and challenges. Indian J Public Health 2011;55: 220–227. 10.4103/0019-557X.89941 22089690

[12] World Health Organisation. WHO Report on the Global Tobacco epidemic, 2013: Enforcing bans on tobacco advertising, promotion and sponsorship © World Health Organisation 2013 Available at: http://www.who.int/tobacco/global_report/2013/en/index.html. Last accessed: August 2014

[13] Planning Commission, Government of India. Eleventh Five Year Plan (2007–2012) Volume II: Social sector. Oxford University Press, New Delhi, 2008 Available at: http://planningcommission.nic.in/plans/planrel/fiveyr/11th/11_v2/11th_vol2.pdf. Last accessed: August 2014.

[14] Planning Commission, Government of India. Twelfth Five Year Plan (2012–2017) Volume III: Social sectors. SAGE Publications, New Delhi, 2013 Available at: http://planningcommission.nic.in/plans/planrel/fiveyr/12th/pdf/12fyp_vol3.pdf. Last accessed: August 2014.

[15] World Health Organization, Centers for Disease Control and Prevention, and the Canadian Public Health Association. Global Tobacco Surveillance System Data (GTSSData), 2013. Available at: http://nccd.cdc.gov/GTSSData/default/default.aspx. Last updated: January 2013. Accessed 2014 August.

[16] Kashiwabara M, Arul R, Goswami H, Narain JP, Armada F. Local governments and civil society lead breakthrough for tobacco control: Lessons from Chandigarh and Chennai. Indian J Public Health 2011;55: 234–239. 10.4103/0019-557X.89937 22089692

[17] Bhatia V, Puri S, Kaur A, Mayank V. Impact of ban of smoking in first smoke free city of Chandigarh in India. 14^th^ World Conference on Tobacco or Health. Mumbai, India, 2009.

[18] John RM, Glantz SA. It is time to make smoke-free environments work in India. Indian J Med Res 2007;125: 599–603. 17642492

[19] Arora M, Stigler MH, Reddy KS. Effectiveness of health promotion in preventing tobacco use among adolescents in India: Research evidence informs National Tobacco Control Programme in India. Glob Health Promot 2011;18: 9–12. 21721292 10.1177/1757975910393163 PMC3132087

[20] Flay BR. The promise of long-term effectiveness of school-based smoking prevention programs: a critical review of reviews. Tob Induc Dis 2009;5: 7 10.1186/1617-9625-5-7 19323827 PMC2669058

[21] Moher D, Liberati A, Tetzlaff J, Altman DG, The PRISMA Group. Preferred Reporting Items for Systematic Reviews and Meta-Analyses: The PRISMA Statement. PLoS Med 2009;6: e1000097 Available at: 10.1371/journal.pmed.1000097. Accessed 2014 Aug. 10.1371/journal.pmed.1000097 19621072 PMC2707599

[22] Centre for Reviews and Dissemination. Systematic Reviews: CRD’s guidance for undertaking reviews in health care © Centre for Reviews and Dissemination, University of York, 2008 [Available: http://www.york.ac.uk/inst/crd/pdf/Systematic_Reviews.pdf]. Accessed 2014 Aug.

[23] Effective Public Health Practice Project. Quality assessment tool for quantitative studies. Available at: http://www.ephpp.ca/tools.html. Accessed 2014 Aug.

[24] The Cochrane Collaboration. Cochrane Handbook for Systematic Reviews of Interventions, 2011. Available at: http://www.cochrane-handbook.org/. Date last updated: March 2011. Accessed 2014 Aug.

[25] Singh V, Sharma BB, Saxena P, Meena H, Mangal DK. Price and consumption of tobacco. Lung India 2012;29: 203–206. 10.4103/0970-2113.99095 22919157 PMC3424857

[26] Murugaboopathy V, Ankola AV, Hebbal M, Sharma R. Indian dental students' attitudes and practices regarding tobacco cessation counseling. J Dent Educ 2013;77: 510–517. 23576597

[27] Rajasundaram P, Sequeira PS, Jain J. Perceptions of dental students in India about smoking cessation counseling. J Dent Educ. 2011;75: 1603–1610. 22184600

[28] Sinha DN, Rinchen S, Palipudi KM, Naing Shein N, de Silva P, Khadka BB, et al Tobacco use, exposure to second-hand smoke, and cessation training among the third-year medical and dental students in selected Member States of South-East Asia region: A trend analysis on data from the Global Health Professions Student Survey, 2005–2011. Indian J Cancer 2012;49: 379–386. 10.4103/0019-509X.107743 23442402

[29] Sinha DN, Gupta PC, Reddy KS, Prasad VM, Rahman K, Warren CW, et al Linking Global Youth Tobacco Survey 2003 and 2006 data to tobacco control policy in India. J School Health 2008;78: 368–373. 10.1111/j.1746-1561.2008.00316.x 18611211

[30] Sinha DN, Gupta PC. Tobacco control practices by medical doctors in developing world; a questionnaire study. Indian J Public Health 2004;48:144–146. 15709602

[31] Zahiruddin QS, Gaidhane A, Bawankule S, Nazli K, Zodpey S. Prevalence and pattern of tobacco use among tribal adolescents: Are tobacco prevention messages reaching the tribal people in India. Ann Trop Med Public Health 2011;4: 74–80.

[32] Dongre A, Deshmukh P, Murali N, Garg B. Tobacco consumption among adolescents in rural India: where and how tobacco control should focus its attention? Indian J Cancer 2008;45: 100–106. 19018113 10.4103/0019-509x.44065

[33] Sorensen G, Gupta PC, Sinha DN, Shastri S, Kamat M, Pednekar MS, et al Teacher tobacco use and tobacco use prevention in two regions in India: results of the Global School Personnel Survey. Prev Med. 2005;41: 417–423. 15917036 10.1016/j.ypmed.2004.09.048

[34] Sinha DN, Gupta PC, Warren CW, Asma S. School policy and tobacco use by students in Bihar, India. Indian J Public Health 2004;48: 118–122. 15709597

[35] Pednekar MS, Gupta PC. Tobacco use among school students in Goa, India. Indian J Pub Health 2004;48: 147–152. 15709603

[36] Murukutla N, Turk T, Prasad CV, Saradhi R, Kaur J, Gupta S, et al Results of a national mass media campaign in India to warn against the dangers of smokeless tobacco consumption. Tob Control 2012;21: 12–17. 10.1136/tc.2010.039438 21508418

[37] Thresia CU, Thankappan KR, Nichter M. Smoking cessation and diabetes control in Kerala, India: an urgent need for health education. Health Educ Res. 2009;24:839–845. 10.1093/her/cyp020 19332439 PMC2738957

[38] Sansone GC, Raute LJ, Fong GT, Pednekar MS, Quah ACK, Bansal-Travers M, et al Knowledge of health effects and intentions to quit among smokers in India: findings from the Tobacco Control Policy (TCP) India pilot survey. Int J Environ Res Public Health. 2012;9: 564–578. 10.3390/ijerph9020564 22470310 PMC3315264

[39] Raute LJ, Sansone G, Pednekar MS, Fong GT, Gupta PC, Quah AC, et al Knowledge of health effects and intentions to quit among smokeless tobacco users in India: findings from the International Tobacco Control Policy Evaluation (ITC) India Pilot Survey. Asian Pac J Cancer Prev. 2011;12: 1233–1238. 21875273

[40] Sharma I, Sarma PS, Thankappan KR. Awareness, attitude and perceived barriers regarding implementation of the Cigarettes and Other Tobacco Products Act in Assam, India. Ind J Cancer 2010;47:63–68. 10.4103/0019-509X.63874 20622417

[41] Tiwari RR, Zodpey SP. Use of smokeless tobacco—a community-based study of behaviour, attitudes and beliefs. Regional Health Forum WHO South-East Asia Region 2006;3: 1–4.

[42] Sharma R, Pednekar MS, Rehman AU, Gupta R. Tobacco use among school personnel in Rajasthan, India. Indian J Cancer 2004;41: 162–166. 15659869

[43] Sinha DN, Gupta PC. Tobacco use among school personnel in Orissa. Indian J Public Health 2004;48: 123–127. 15709598

[44] Sinha DN, Roychowdhury S. Tobacco control practices in 25 schools of West Bengal. Indian J Public Health 2004;48: 128–131. 15709599

[45] Sinha DN, Gupta PC. Tobacco use among students in Uttar Pradesh and Uttaranchal, India. Indian J Public Health 2004;48: 132–137. 15709600

[46] Halawany HS, Jacob V, Abraham NB, Al-Maflehi. Oral cancer awareness and perception of tobacco use cessation counselling among dental students in four Asian countries. Asian Pacific J Cancer Prev 2013;14: 3619–3623. 23886155 10.7314/apjcp.2013.14.6.3619

[47] Panda R, Jena PK. Examining physicians’ preparedness for tobacco cessation services in India: findings from primary care public health facilities in two Indian states. Australas Med J 2013;6: 115–121. 10.4066/AMJ.2013.1617 23589736 PMC3626027

[48] Thankappan KR, Yamini TR, Mini GK, Arthur C, Sairu P, Leelamoni K, et al Assessing the readiness to integrate tobacco control in medical curriculum: experiences from five medical colleges in southern India. Nat Med J India 2013;26: 18–23. 24066988

[49] Mony PK, Jayakumar S. Preparedness for tobacco control among postgraduate residents of a medical college in Bangalore. Indian J Community Med. 2011;36: 104–108. 10.4103/0970-0218.84127 21976793 PMC3180933

[50] Thankappan KR, Pradeepkumar AS, Nichter M. Doctors' behaviour & skills for tobacco cessation in Kerala. Indian J Med Res 2009;129: 249–255. 19491416

[51] Bedi RS. The General Practitioner And The Anti—Tobacco Campaign—A Survey In Punjab. Lung India 1995;13: 51–82.

[52] Notani PN. The role of general medical practitioners in tobacco control programmes: a study in Bombay, India. Health Educ Res 1991;6:121–124. 10148729 10.1093/her/6.1.121

[53] Arora M, Mathur N, Gupta VK, Nazar GP, Reddy KS, Sargent JD. Tobacco use in Bollywood movies, tobacco promotional activities and their association with tobacco use among Indian adolescents. Tob Control 2011;21: 482–487. 10.1136/tc.2011.043539 21730099 PMC3420563

[54] Arora M, Reddy KS, Stigler MH, Perry CL. Associations between tobacco marketing and use among urban youth in India. Am J Health Behav. 2008;32: 283–294. 18067468 10.5555/ajhb.2008.32.3.283 PMC2830491

[55] Sinha DN, Gupts PC, Pednekar M. Tobacco use among students in Bihar (India). Indian J Public Health 2004;48: 111–117. 15709596

[56] Chandrashekar J, Manjunath BC, Unnikrishnan M. Addressing tobacco control in dental practice: a survey of dentists' knowledge, attitudes and behaviours in India. Oral Health Prev Dent. 2011;9: 243–249. 22068180

[57] Srivastava S, Malhotra S, Harries AD, Lal P, Arora M. Correlates of tobacco quit attempts and cessation in the adult population of India: secondary analysis of the Global Adult Tobacco Survey, 2009–10. BMC Public Health 2012;13: 263–270. 10.1186/1471-2458-13-263 PMC3614880 23521839

[58] Pradeepkumar AS, Thankappan KR, Nichter M. Smoking among tuberculosis patients in Kerala, India: proactive cessation efforts are urgently needed. Int J Tuberc Lung Dis 2008;12: 1139–1145. 18812043

[59] Sorensen G, Gupta PC, Nagler E, Viswanath K. Promoting life skills and preventing tobacco use among low-income Mumbai youth: effects of Salaam Bombay Foundation intervention. PLoS ONE 2012;7: e34982 10.1371/journal.pone.0034982 22523567 PMC3327682

[60] Brown HS 3rd, Stigler M, Perry C, Dhavan P, Arora M, Reddy KS. The cost-effectiveness of a school-based smoking prevention program in India. Health Promot Int 2013;28: 178–186. 10.1093/heapro/dar095 22271928 PMC3651691

[61] Stigler MH, Perry CL, Smolenski D, Arora M, Reddy KS. A mediation analysis of a tobacco prevention program for adolescents in India: how did project MYTRI work? Health Educ Behav 2011;38: 231–40. 10.1177/1090198110372330 21411716 PMC3096703

[62] Perry CL, Stigler MH, Arora M, Reddy KS. Preventing tobacco use among young people in India: Project MYTRI. *Am J Public Health* 2009;99: 899 *–* 906. 19299670 10.2105/AJPH.2008.145433 PMC2667859

[63] Goenka S, Tewari A, Arora M, Stigler MH, Perry CL, Arnold JP, et al (2010) Process evaluation of a tobacco prevention program in Indian schools—methods, results and lessons learnt. Health Educ Res. 2010;25: 917–935. 10.1093/her/cyq042 20884731 PMC3003490

[64] Bate SL, Stigler MH, Thompson MS, Arora M, Perry CL. Psychosocial mediators of a school-based tobacco prevention program in India: results from the first year of project MYTRI. Prev Sci 2009;10: 116–28. 10.1007/s11121-008-0113-x 19023657 PMC2821665

[65] Reddy KS, Arora M, Perry CL, Nair B, Kohli A, Lytle LA, et al. Tobacco and alcohol use outcomes of a school-based intervention in New Delhi. *Am J Health Behav* 2002;26: 173 *–* 181. 12018753 10.5993/ajhb.26.3.2

[66] Arora M, Tewari A, Tripathy V, Nazar GP, Juneja NS, Ramakrishnan L, et al Community-based model for preventing tobacco use among disadvantaged adolescents in urban slums of India. Health Promot Int. 2010;25: 143–152 10.1093/heapro/daq008 20190265

[67] Singh P, Kumar R. Assessment of the effectiveness of sustained release Bupropion and intensive physician advice in smoking cessation. Lung India 2010;27: 1–38. 10.4103/0970-2113.59259 20539765 PMC2878706

[68] Thankappan KR, Mini GK, Daivadanam M, Vijayakumar G, Sumar PS, Nichter M. Smoking cessation among diabetes patients: results of a pilot randomized controlled trial in Kerala, India. BMC Public Health 2013;13: 47–53. 10.1186/1471-2458-13-47 23331722 PMC3560246

[69] Kumar MS, Sarma PS, Thankappan KR. Community-based group intervention for tobacco cessation in rural Tamil Nadu, India: a cluster randomized trial. J Subst Abuse Treat 2012;43: 53–60. 10.1016/j.jsat.2011.10.026 22154037

[70] Resource Centre for Tobacco Free India. Government of India initiatives [website] © 2007 Resource Centre for Tobacco Free India Available at: http://rctfi.org/goi_initiatives.htm. Last accessed: August 2014

[71] Mohanty VR, Rajesh GR, Aruna DS. Role of dental institutions in tobacco cessation in India: current status and future prospects. Asian Pacific J Cancer Prev 2013;14: 2673–2680. 23725194 10.7314/apjcp.2013.14.4.2673

[72] Resource Centre for Tobacco Free India. Government of India initiatives: National programme for tobacco control during 11th five year plan proposed—(2007–2012) [website] © 2007 Resource Centre for Tobacco Free India Available at: http://rctfi.org/goi_initiatives5.htm. Accessed 2014 Aug.

[73] Goswami H, Kashyap R. Tobacco in movies and impact on youth Chandigarh, India, Burning Brain Society, 2006 Available at: http://smokefreemovies.ucsf.edu/pdf/BurningBrain-tobaccoinmovies.pdf. Accessed 2014 Aug.

[74] Mekernson C, Glantz SA. How the tobacco industry built its relationship with Hollywood. Tob Control 2002;11(s1):i81–i91. 11893818 10.1136/tc.11.suppl_1.i81 PMC1766059

[75] World Health Organization. Smoke-free movies: from evidence to action (2nd ed.) © World Health Organization 2011 Available at: http://whqlibdoc.who.int/publications/2011/9789241502399_eng.pdf?ua = 1. Accessed 2014 Aug.

[76] Basu S, Glantz S, Bitton A, Millett C. The Effect of Tobacco Control Measures during a Period of Rising Cardiovascular Disease Risk in India: A Mathematical Model of Myocardial Infarction and Stroke. PLoS Med 2013;10: e1001480 10.1371/journal.pmed.1001480 23874160 PMC3706364

[77] Stead LF, Lancaster T. Interventions to reduce harm from continued tobacco use. Cochrane Database Syst Rev 2007;3: CD005231 17636791 10.1002/14651858.CD005231.pub2

[78] Hughes JR, Keely J, Naud S. Shape of the relapse curve and long-term abstinence among untreated smokers. Addiction 2004;99: 29–38. 14678060 10.1111/j.1360-0443.2004.00540.x

[79] Public Health Foundation of India. Short-term courses on tobacco control, 2013 [brochure]. Available at: http://www.phfi.org/images/institute/stcbrochure_2013_final.pdf. Accessed 2014 Aug.

