# Supplementary material for: Strategies for Tobacco Control in India: A Systematic Review
Source: PLoS One. 2015 Apr 9;10(4):e0122610. doi: 10.1371/journal.pone.0122610 (PMC4391913; doi:10.1371/journal.pone.0122610)
Supplement: S1 File — Table A in S1 File—Studies related to FCTC articles 6, 11, 13 and 16. Reviewed studies relating to FCTC articles 6, 11, 13 and 16 excluded from analysis of outcomes. The numbers following the different quality categories (SA, US, NA) indicate the aspect of quality assessment (see Table 2), rated as satisfactory (SA), unsatisfactory (US) or not-assessable (NA). The main reasons for concerns regarding study reliability are also listed. All studies were of cross-sectional design. * = part of study included in analysis of outcomes. U = urban; R = rural; FGD = focus group discussion; NR = not reported; COTPA = Cigarette and Other Tobacco Products Act; LSGB = local self-government bodies; S = significant; NS = not significant. Table B in S1 File—Studies related to FCTC Article 8: Protection from exposure to tobacco smoke. Reviewed studies relating to FCTC Article 8 excluded from analysis of outcomes. The numbers following the different quality categories (SA, US, NA) indicate the aspect of quality assessment (see Table 2), rated as satisfactory (SA), unsatisfactory (US) or not-assessable (NA). The main reasons for concerns regarding study reliability are also listed. All studies were of cross-sectional design. * = part of study included in analysis of outcomes. NR = not reported; U = urban; R = rural; LSGB = local self-government bodies; SHS = second-hand smoke; COTPA = Cigarette and Other Tobacco Products Act; FGD = focus group discussion; S: significant; NS: non-significant. Table C in S1 File—Studies related to FCTC Article 12: Education, communication, training and public awareness. Reviewed studies relating to FCTC Article 12 excluded from analysis of outcomes, by sub-category. The numbers following the different quality categories (SA, US, NA) indicate the aspect of quality assessment (see Table 2), rated as satisfactory (SA), unsatisfactory (US) or not-assessable (NA). The main reasons for concerns regarding study reliability are also listed. All studies were of cr [file pone.0122610.s002.docx]

**Table A – Studies related to FCTC articles 6, 11, 13 and 16**

| **Ref. (year of publication)** | **Study dates & location** | **Sample size & characteristics** | **Tobacco use prevalence** | **Methods** | **Main outcome measures (bold) & results** | **Quality assessment (numerical ratings and main concerns)** |
| --- | --- | --- | --- | --- | --- | --- |
| **Article 6: Price and tax measures to reduce the demand for tobacco** | | | | | | |
| s1 (2008)* | 2008; Wardha (R) | n = 6-8 per FGD; 15–19 years; 100 % male | NR | 6 x FGDs | Tobacco purchased with pocket money or wages, in some cases purchased as part of family groceries | SA: 1-2, 11; US: 3-5, 7-10; NA: 6 (Sampling, aims, extent of data collection & intended analysis unclear, risk of recall bias) |
| s2 (2010)* | 2005; Delhi (U) | 5 FGDs with n = 37 (total); residents of 2 low-SES communities; 10–19 years; 83.8 % male; high prevalence of other addictions | NR | FGDs | Tobacco offered from friends/family; tobacco easily available, money generally not barrier as cheap products available, most find difficult to resist peer influence | SA: 1, 2; NS: 8-11; NA: 3- 7 (Low n-number; recruitment, data collection & analysis unclear; risk of measurement bias) |
| **Article 11: Packaging and labeling of products** | | | | | | |
| s3 (2012) | 2011; Odisha (mainly U) | n = 1414; mean age 35.1+11.6 years; 70.6 % male | 47.2 % of those 31–40 years, 55.1 % 41–50 years, 60.6 % > 50 years | Interview regarding COPTA provisions | 66.6 % never seen **mandatory warning signs at point of sale**, 36.2 % never seen **pictorial warnings on packaging**, 84.3 % seen **mandatory warnings in English**, 58.3 % in **Hindi**, 37.1 % in **local language** | SA: 1,7; US: 2, 10, 11; NA: 3-6,8,9 (Aims, sampling, intended data collection & analysis unclear; non-random sampling; potential recall bias) |
| s4 (2013) | 2010; Mumbai (U) | Study of designated slum area in Mumbai | NR | Observational techniques | 68 **tobacco products available**, all packed **products displayed warnings**, but loose tobacco did not. Approx. ½ of packs did not **list contents** in English or Hindi. | SA: 1,11; US: 2,10; NA: 3-9 (Study design, aims, methods & analysis unclear) |
| s5 (2012) | 2009; Delhi, Uttarakhand, Haryana, Uttar Pradesh, Tripura (U) | n= 1897; 10–50 years; 56 % male | 54 % tobacco users | Interview one month post- introduction of pictorial warnings on tobacco packs | 64.47 % **considered warnings inadequate to convey health impact**, 52.92 % **felt they would not motivate users to quit**; | SA: 1, 11; US: 2, 7, 10; NA: 3- 6, 8, 9 (Aims, recruitment & interview methods unclear, analysis incompletely reported, potential for recall bias) |
| s6 (2011) | 2009; Mumbai (U) | n = 615; > 18 years; 74.6 % male; | 59.2 % ever tobacco users | 4 x FGDs & questionnaire survey | Males/females: had **read warnings on smokeless products**: 53.2 %/23.1 %, **& smoked products**: 62.7 %/26.3 %; had **seen pictorial health warning on smoked products**: 68.8 %/30.8 %, **& smokeless products:** 76%/47.4 %; little understanding of image of lungs, scorpion 'conveyed some message' | SA: 1; US: 2, 7, 9-11; NA: 3- 6, 8 (No controls; aims, recruitment & extent of analysis unclear, intended/reported outcomes inconsistent, duration of intervention unclear) |
| s7 (2009) | Dates NR; Mumbai, Thane (U) | n = 712; 15 to> 55 years; 64.5 % male | 70.2 % never tobacco users, 28.2 % current users | Interview | **Aware of existing tobacco packaging pictorial warnings**: 89.6 %; **as result of warnings**: 23.2 % male users **considered quitting**, 36.7 % **considered reducing use**, 33.1 % **never users would re-consider before taking-up** | SA: 1, 11; US: 2, 7, 9, 10; NA: 3-6, 8 (Convenience sample; no controls; aims, recruitment & intended analyses unclear; additional outcomes reported; conclusions not directly linked to data collected) |
| s8 (2007) | 2005; Tamil Nadu (R) | n = 15 interviewees & 7-8 per FGD; 66 % of interviewees male; all > 10 years (in FGDs females > 50, males < 50 years) | 100 % areca nut users | (a) Interviews, (b) FGDs x 5 | No **advertisements on ill-effects of areca nut products identified**; no **health warnings apparent ‘in communities visited’** (NB. details of visits NR) | SA: 1; US: 2,3,5,8,10,11; NA: 4,6,7,9 (Sampling unclear, small n-numbers, different age groups for males/females, data collection & analysis unclear) |
| **Article 13: Tobacco advertising, promotion and sponsorship** | | | | | | |
| s4 (2013) | 2010; Mumbai (U) | Study of designated slum area in Mumbai | NR | Observational techniques | **Marketing & promotional activities**: creative marketing noted at outlets, plus various promotions & surrogate advertising | SA: 1,11; US: 2, 10; NA: 3-9 (Study design, aims, methods & analysis unclear) |
| s9 (2013) | Dates NR; Kerala (67.7 % R) | n = 956; LSGB representatives; mean age 44+10.7 years; 59.8 % male | 14.9 % (0.8 % of females) | Interviews | Regional **ban** **on advertising in print media & outdoor spaces**: 14.1 % | SA: 1, 11; US: 2, 7, 9, 10; NA: 3-6, 8 (Sampling & data collection methods NR, data collected & analysis unclear) |
| s10 (2012) | 2009; Mumbai (U) | n = 534; school students; 9–16 years; sex NR | Ever use: 5.1 %; current use: 1 %; ever areca nut use: 2.6 % | Questionnaire | 3 % **intention to quit**: S **association with exposure to marketing/ promotions** (p = 0.007) | SA: 1, 2, 11; US: 8-10; NA: 3- 7 (School recruitment & data collected unclear, intended analysis NR, retrospective analysis, evidence of additional outcomes) |
| s11 (2006) | Dates NR; Rajasthan (U) | n = 3805; school students; 13–18 years; 75.3 % male | Males/females: smokers: 1.5 %/0.85 %; smokeless users: 0.56 %/0.85 % | NR | 55.5 % boys & 54.0 % girls **remembered advertisement & could name tobacco product** | SA: 1, 2; US: 7-11; NA: 3-6 (Recruitment methods, data collection intentions & methods NR; intended comparisons unclear) |
| **Article 16: Sale to-and-by minors** | | | | | | |
| s4 (2013) | 2010; Mumbai (U) | Study of designated slum area in Mumbai | NR | Observational & spatial field research | Most developed community had highest **density of outlets**; v**iolations of legislation** noted, including outlets within 100 m of schools & religious institutions | SA: 1,11; US: 2,10; NA: 3-9 (Study design, aims, methods & analysis unclear) |
| s9 (2013) | Dates NR; Kerala (67.7 % R) | n = 956; LSGB representatives; mean age 44+10.7 years; 59.8 % male | 14.9 % (0.8 % of females) | Interviews | **Regional ban on sale to minors**: 44 % | SA: 1, 11; US: 2, 7, 9, 10; NA: 3-6, 8 (Sampling & data collection methods NR, data collected & analysis unclear) |
| s3 (2012) | 2011; Odisha (mainly U) | n = 1414; non-random sample; mean age 35.1+ 11.6 years; 70.6 % male | 47.2 % of those 31–40 years, 55.1 % 41–50 years, 60.6 % > 50 years | Interview regarding COPTA provisions | 36.6 % **‘very often’ see tobacco sold to minors**, 24.8 % **‘very often’ see** **tobacco sold within 100 yards of education institution**, 77.7 % never seen **signs prohibiting sale near education institutions** | SA: 1,7; US: 2, 10, 11; NA: 3-6,8,9 (Aims, sampling, intended data collection & analysis unclear; non-random sampling; potential recall bias) |
| s8 (2007) | 2005; Tamil Nadu (R) | n = 15 interviewees & 7-8 per FGD; 66 % of interviewees male; all > 10 years (in FGDs females > 50, males < 50 years) | 100 % areca nut users | (a) Interviews, (b) FGDs x 5 | Minors have no difficulty purchasing areca nut products with tobacco | SA: 1; US: 2,3,5,8,10,11; NA: 4,6,7,9 (Sampling unclear, small n-numbers, different age groups for males/females, data collection & analysis unclear) |

Reviewed studies relating to FCTC articles 6, 11, 13 and 16 excluded from analysis of outcomes. The numbers following the different quality ‘levels’ (SA, US, NA) indicate the aspect of quality assessment (see Box 1), rated as satisfactory (SA), unsatisfactory (US) or not-assessable (NA), and the main reasons for concerns regarding study reliability also listed. All studies were of cross-sectional design. * = parts of study included in analysis of outcomes

U = urban; R = rural; FGD = focus group discussion; NR = not reported; COTPA = Cigarette and Other Tobacco Products Act; LSGB = local self-government bodies; S = significant; NS = not significant

**Table B – Studies related to FCTC Article 8: Protection from exposure to tobacco smoke**

| **Ref (year of publication)** | **Study dates & location** | **Sample size & characteristics** | **Tobacco use prevalence** | **Methods** | **Main outcome measures (bold) & results** | **Quality assessment (numerical ratings and main concerns)** |
| --- | --- | --- | --- | --- | --- | --- |
| s9 (2013) | Dates NR; Kerala (67.7 % R) | n =956; LSGB representatives; mean age 44+10.7 years; 59.8 % male | 14.9 % (0.8 % of females) | Interviews | Frequent **SHS exposure**: 66 %; regional **ban on** **smoking in public places:** 46.9 %, & **workplaces**: 46.2 % | SA: 1, 11; US: 2, 7, 9, 10; NA: 3-6, 8 (Sampling & data collection methods NR, data collected & analysis unclear) |
| s3 (2012) | 2011; Odisha (mainly U) | n = 1414; non-random sample; mean age 35.1+ 11.6 years; 70.6 % male | 47.2 % of those 31–40 years, 55.1 % 41–50 years, 60.6 % > 50 years | Interview regarding COPTA provisions | 24.9 % **aware regional control programmes being implemented**; aware of **‘smoke free’ areas in practice:** 3 %; **items to facilitate smoking in public places** ‘often’ seen | SA: 1,7; US: 2, 10, 11; NA: 3-6,8,9 (Aims, sampling, intended data collection & analysis unclear; non-random sampling; potential recall bias) |
| s12 (2011) | 2009; Mumbai (U) | n = 5 restaurants, 4 pubs, 27 ‘bars & restaurants’, 10 country liquor bars, 4 hookah restaurants | n/a | Observational study, air quality monitoring, interaction with staff | **Smoking observed in venues**: 18/50, **‘no smoking’ signs in venues:**  33/50; **air quality index and category of health concern**: average air quality in ‘restaurants & bars’ of ‘moderate’ health concern, ‘hazardous level’ at country liquor bars & hookah restaurants | SA: 1,2,11; US: 6,7,9,10; NA: 3-5,8 (Venue selection & location unclear, small n-numbers, no control for other potential pollutants, outcomes incompletely reported, implications of outcomes unclear) |
| s13 (2011) | 2008-09; Ahmedabad, Chandigarh, Chennai, Delhi (U) | n = between 4 & 10 for each type of establishment (bars, government building, hospital, restaurant, school) in each city | n/a | Observational survey, nicotine sampling, staff questionnaire | **Median, low & high air nicotine concentrations reported for each type of establishment** in each city. | SA: 1,2,10,11; US: 4,6-8; NA: 3,5,9 (Convenience sample, controls unclear, some outcomes NR, implications of results unclear) |
| s14 (2011) | Dates NR; Sikkim (U+R), Shimla (U), Coimbatore (U), Villupuram area (semi-U) | Four regions declared smoke-free in 2010, where enforcement capacity & strategy developed, & systematic monitoring of compliance with smoke-free legislation implemented | n/a | Observational survey | Across all sites: > 97 % no **active smoking**, 82-97 % **signs displayed**; 87-98 % no **tobacco smoke detected**; 88-97 % no articles to facilitate smoking, 86-98 % no cigarette waste | SA: 1,4,5,8; US: 2,10,11; NA: 3,6,7,9 (Limited details of intervention and methods reported, no controls) |
| s15 (2009) | 2008; Chandigarh (U) | n = 209 smokers; 18 tobacco vendors; 18 - > 45 years; sex NR | NR for vendors, otherwise 100 % smokers | Interview at almost 1 year post imposition of smoking ban | **Aware of ban**: 70.3 % ‘participants', 72.2 % vendors; **decline in sale**: 55.5 % of vendors; **source of information about ban:** 67.9 % friends, 22 % newspaper, 22 % other media; 6.2 % family; **no difficulty buying cigarettes post-ban**: 86.1 %; **reduced frequency of smoking**: 19.6 % of smokers, 20.6 % of their friends | SA: n/a; US: 1, 2, 10, 11; NA: 3-9 (Aims & methods unclear, no analysis, no controls) |
| s11 (2006) | Dates NR; Rajasthan (U) | n = 3805, school students; 13 – 18 years; 75.3 % male | Boys/girls: 1.5 %/0.85 % smokers; 0.56 %/0.85 % smokeless users | NR | **Tobacco use in immediate family**: 42.1 % males, 32.4 % females; **previous attempt at tobacco control among family/peers**: 5.2 % | SA: 1, 2; US: 7-11; NA: 3-6 (Recruitment methods, data collection intentions and methods NR; intended comparisons unclear) |
| s16 (2005) | 2002; Delhi (U) | n = 435; school students; 10–16 years; 58.4 % male | NR | FGD | Generally irritated by adults smoking around them; parents responses to being asked to stop varied | SA: 1; US: 2, 6, 8-11; NA: 3-5,7 (School selection unclear; small number of schools; analysis limited, risk of measurement bias) |
| s17 (2004) | 2000-01; Bihar (U+R) | State/federal school personnel: n = 637/1638; age & sex NR  (NB. State schools surveyed in 2000, federal schools in 2001) | State/federal school personnel: daily smokers: 14.5%/2.4 %, daily smokeless tobacco users: 41.7 %/14.1 % | Self-administered questionnaire | Federal/state schools: **policy prohibiting use among students**: 53.4 %/0.2 %, **& staff**: 67.3 %/0.1 %; **policy enforced**: 47.1 %/0.1 % | SA: 1,4,5,8; US: 2,3,6,7,9-11; NA: n/a (Federal schools surveyed after state schools, potential confounding factors (e.g. S more tobacco use in state schools), data collected & intended analysis unclear) |
| s18* (2004) | 2000 (state schools), 2001 (federal schools); Bihar (U+R) | n = 2636 (state schools), 3951 (federal schools); school students; 13–15 years; sex NR | Ever tobacco use: 72.8 % R state schools, 35.6 % R federal schools, 70.0 % U state, 35.2 % U federal | Self-administered questionnaire | Parental tobacco use similar amongst students attending state & federal schools | SA: 1, 3-5, 8; US: 2, 10, 11; NA: 6, 7, 9 (Aims of data collection & analysis NR, no sizes of differences or statistics reported) |

Reviewed studies relating to FCTC Article 8 excluded from analysis of outcomes. The numbers following the different quality ‘levels’ (SA, US, NA) indicate the aspect of quality assessment (see Box 1), rated as satisfactory (SA), unsatisfactory (US) or not-assessable (NA), and the main reasons for concerns regarding study reliability also listed. All studies were of cross-sectional design. * = parts of study included in analysis of outcomes

NR = not reported; U = urban; R = rural; LSGB = local self-government bodies; SHS = second-hand smoke; COTPA = Cigarette and Other Tobacco Products Act; FGD = focus group discussion; S: significant; NS: non-significant

**Table C – Studies related to FCTC Article 12: Education, communication, training and public awareness**

| **Ref. (year of publication)** | **Study dates & location** | **Sample size & characteristics** | **Tobacco use prevalence** | **Methods** | **Main outcome measures (bold) & results** | **Quality assessment (numerical ratings and main concerns)** |
| --- | --- | --- | --- | --- | --- | --- |
| **Young people** | | | | | | |
| s10 (2012) | 2009; Mumbai (U) | n = 534; school students; 9–16 years; sex NR | 5.1 % ever tobacco users, 1 % current users | Questionnaire | 3 % **intention to quit**: no **association with exposure to tobacco-related messages** | SA: 1, 2, 11; US: 8-10; NA: 3- 7 (School recruitment & data collected unclear, intended analysis NR, retrospective analysis, evidence of additional outcomes) |
| s1 (2008)* | 2008; Wardha (R) | n = 6-8 per FGD; 15–19 years; 100 % male | NR | 6 x FGDs | Tobacco used for fatigue & several medical problems | SA: 1-2,11; US: 3-5, 7-10; NA: 6 (Sampling, aims, extent of data collection & intended analysis unclear, risk of recall bias) |
| s2 (2010)* | 2005; Delhi (U) | n = 37; low-SES; 10–19 years; 83.8 % male; high prevalence of addictions | NR | FGDs | ‘Almost all’ consider **tobacco injurious to health**; most aware of some specific consequences, but unable to **distinguish long-/short-term consequences;** most **aware of Tobacco Control Act** | SA: 1,2; US: 8-11; NA: 3-7 (Low n-number; recruitment NR; data collection & analysis unclear; risk of measurement bias) |
| s11 (2006) | Dates NR; Rajasthan (U) | n = 3805; school students; 13–18 years; 75.3 % male | Males/ females: 1.5 %/0.85 % smokers; 0.56 %/0.85 % smokeless users | NR | **Aware tobacco harmful**: 99.2 % males, 99.5 % females**; aware of link with various disease**s: 23.2 - 96.5 % (variation by disease); **consider quitting beneficial**: 76.4 % males, 75.7 % females | SA: 1, 2; US: 7-11; NA: 3- 6 (Recruitment methods, data collection intentions & methods NR; intended comparisons unclear) |
| s19 (2005) | 2003-04; Maharashtra & Bihar (U+R) | n = 139; school teachers; age, sex NR | NR | FGDs x 12 | Recognising tobacco use in students not priority issue; smokeless use considered less serious than smoking; tobacco education limited as little attention in national curriculum, time constraints & query as to need; school anti-tobacco policies generally supported; expression of need for these to form part of broader efforts including parent education | SA: 1, 2, 7, 10; US: 5, 6, 8, 9, 11; NA: 3, 4 (Recruitment unclear, data collection via FGDs, therefore ambiguity in measurement & outcomes) |
| s16 (2005) | 2002; Delhi (U) | n = 435; school students; 10–16 years; 58.4 % male | NR | FGDs | Several **negative health consequences of tobacco use understood**; general uncertainty regarding benefits of quitting, impression withdrawal should be gradual | SA: 1; US: 2, 6, 8-11; NA: 3-5, 7 (School selection unclear; small number of schools; analysis limited, risk of measurement bias) |
| s17 (2004) | 2000-01; Bihar (U+R) | State/federal school personnel: n = 637/1638; age & sex NR  (NB. State schools surveyed in 2000, federal schools in 2001) | State/federal school personnel: daily smokers: 14.5%/2.4 %, daily smokeless tobacco users: 41.7 %/14.1 % | Self-administered questionnaire | **Students taught about long-term effects of tobacco**: 44.1 %/0.5 %, **& short-term effects**: 58.4 %/0.6 %; **relevant teacher training received**: 6.7 %/1 %, **access to teaching materials**: 35.0 %/1.2 % | SA: 1,4,5,8; US: 2,3,6,7,9-11; NA: n/a (Federal schools surveyed after state schools, potential confounding factors (e.g. S more tobacco use in state schools), data collected & intended analysis unclear) |
| **Adults** | | | | | | |
| s9 (2013) | Dates NR; Kerala (67.7 % R) | n = 956; LSGB representatives; mean age 44+10.7 years; 59.8 % male | 14.9 % (0.8 % females) | Interview | **Aware of FCTC**: 17.3 %; **aware of main provisions**: 20.8 – 94.2 % (variation by provision); **one cigarette/day capable of adverse health effects**: 72 % | SA: 1, 11; US: 2, 7, 9, 10; NA: 3-6, 8 (Sampling & data collection methods NR, data collected & analysis unclear) |
| s3 (2012) | 2011; Odisha (mainly U) | n = 1414; mean age 35.1+11.6 years; 70.6 % male | 47.2 % of those 31–40 years, 55.1 % 41–50 years, 60.6 % > 50 years | Interview | **Aware of tobacco control laws**: 52.1 %, **of prohibition of smoking in public places**: 80.8 %, **& associated penalty**: 6.7 %, **aware of regional control programmes**: 24.9 % | SA: 1,7; US: 2, 10, 11; NA: 3-6,8,9 (Aims, sampling, intended data collection & analysis unclear; non-random sampling; potential recall bias) |
| s20 (2010)* | Dates NR; Assam (U) | n = 15; ‘implementers’ of COTPA (including police, teachers, gazetted officers, hotel managers, shop owners), age & sex NR | NR | Interview | Most of those aware of Act unaware of standard specifications; most unaware of own responsibilities, of consequences of Act violation. Most had not received official notification of Act and reported a lack of training. General difficulty interpreting images on tobacco packaging. | SA: 1,2,11 ; US: 8-10 ; NA: 3-7 (Sampling, data collection methods and intentions, and analysis methods unclear; additional outcomes reported) |
| s21 (2009) | 2007–08; Maharashtra (U) | n = 104; chemical industry employees; ‘majority’ > 36 years; sex NR | 48 % | Interview | 100 % aware **tobacco use injurious to health**; 99 % **aware of cancer association;** fewer of **association with other diseases;** 97 % consider **SHS harmful**, 83 % **infrequent tobacco** **use dangerous;** general **belief tobacco will not cause personal harm** | SA: 1,3,4,5,8; US: 2, 7, 9-11; NA: 6 (Aims & extent of data collection unclear, lecture potentially given just prior to interview; analysis more extensive than suggested) |
| s8 (2007) | 2005; Tamil Nadu (R) | n = 15 interviewees & 7-8 per FGD; 66 % of interviewees male; all > 10 years (in FGDs females > 50, males < 50 years) | 100 % areca nut users | (a) Interview (b) FGDs x 5 | **Awareness of health effects**: ‘minimal’; older participants generally believe tobacco not harmful as no problems experienced; some believe paan/gutkha impact on ability to eat/speak | SA: 1; US: 2,3,5,8,10,11; NA: 4,6,7,9 (Sampling unclear, small n-numbers, different age groups for males/females (men > 50 years refused to participate), methods including analysis unclear) |
| s19 (2005) | 2003-04; Maharashtra & Bihar (U+R) | n = 139; school teachers; age, sex NR | NR | FGDs | Generally **awareness tobacco has adverse effects**; uncertainty about **specifics other than cancer**; few **aware of risks of SHS**; more **awareness of effects** of smoking cf. smokeless tobacco; common belief **chewing good for gums/teeth**; **knowledge of formal cessation assistance** low | SA: 1, 2, 7, 10; US: 5, 6, 8, 9, 11; NA: 3, 4 (Recruitment unclear, data collection via FGDs, therefore ambiguity in measurement & outcomes) |
| s22 (2005) | 2003; Chitrakoot (U) | n = 124; 64 % 21–40 years, 26 % > 40 years; 83.1 % male | Current gutka use: 46 %; past gutka use: 34 %; other forms of tobacco use: ‘frequent’ | Interview | 96.0 % **consider gutka harmful**; ‘overall’ suggestion that **gutka contributes to poor oral health, ‘kidney, heart and lung problems’**; some consider gutka helpful | SA: 1; US: 2,4,7,8,10,11; NA: 3,5,6,9 (Convenience sample; inclusion criteria, participation rates, aims & analyses unclear, open-ended questionnaire, therefore difficulty interpreting outcomes, possible reporting bias) |
| **Healthcare professionals** | | | | | | |
| s23 (2010) | Dates NR; Bangalore (U) | n = 100, mean age 36.3+5.32 years, 56 % male | 14 % smokers, 8 % chewing tobacco users | Self-administered questionnaire | **Aware of tobacco link with: cancer**: 100 %, **lung disease:** 89 %; **consider maternal smoking harmful:** 77 %; **aware tobacco psychoactive**: 58 %, & **nicotine most addictive drug**: 33 %; 34 % some **knowledge of behavioural cessation methods**, 74 % **unaware of pharmaceutical methods** | SA: 1,2; US:3,7,10,11; NA:4-6,8,9 (Selection process/inclusion criteria unclear, relevance of enrolment in information session unclear, no details of questionnaire administration) |

Reviewed studies relating to FCTC Article 12 excluded from analysis of outcomes, by sub-category. The numbers following the different quality ‘levels’ (SA, US, NA) indicate the aspect of quality assessment (see Box 1), rated as satisfactory (SA), unsatisfactory (US) or not-assessable (NA), and the main reasons for concerns regarding study reliability also listed. All studies were of cross-sectional design. * = parts of study included in analysis of outcomes

U = urban; R = rural; FGD = focus group discussion; NR = not reported; SES = socioeconomic status; LSGB = local self-government body; FCTC = Framework Convention on Tobacco Control; COTPA = Cigarettes and Other Tobacco Products Act; SHS = second hand smoke

**Table D – Studies related to FCTC Article 14: Demand reduction measures concerning tobacco dependence and cessation**

| **Ref. (year of publication)** | **Study dates & location** | **Sample size & characteristics** | **Tobacco use prevalence** | **Methods** | **Main outcome measures (bold) & results** | **Quality assessment (numerical ratings and main concerns)** |
| --- | --- | --- | --- | --- | --- | --- |
| **Medical/dental practice** | | | | | | |
| s23 (2010) | Dates NR; Bangalore (U) | n = 100, dental surgeons, mean age: 36.3+5.32 years, 56 % male | 14 % smokers, 8 % smokeless tobacco users | Self-administered questionnaire | 52 % **ask patients about tobacco use**, 19 % **actively advocate cessation,** 46 % **follow-up on tobacco use**, 36 % **keep records of tobacco use** | SA:1,2; US:3,7,10,11; NA:4-6,8,9 (Selection process/inclusion criteria unclear, relevance of enrolment in information session unclear, no details of questionnaire administration) |
| s24 (2005) | Dates NR; Orissa (U & R) | n = 720; GPs; age, sex NR | 29 % tobacco users | Interview | 67 % feel **cessation counselling not their role**; 86 % **lack confidence in counselling**, 26 % **uncertain regarding effectiveness of their counselling** | SA: 2; US: 1, 8-11; NA: 3-7 (Abstract; sampling/recruitment, data collection & analysis unclear) |
| **Medical/dental practice: patient surveys** | | | | | | |
| s25 (2013) | Dates NR; Mangalore (U) | n = 175, clinic attendees; 43.4 % < 25 years, 99.4 % male | 100 % smokers | Self-administered questionnaire | **Lack of knowledge regarding smoking cessation**: 51.4 %; **not counseled by doctor/dentist to quit**: 80.6 % (NB. details of data collection unclear, but above responses given as, ‘reasons for not quitting’) | SA: 1,2,6,7; US: 9-11; NA: 3-5, 8 (Sampling, inclusion criteria & data collection unclear, multiple comparisons) |

Reviewed studies relating to FCTC Article 14 excluded from analysis of outcomes, by sub-category. The numbers following the different quality ‘levels’ (SA, US, NA) indicate the aspect of quality assessment (see Box 1), rated as satisfactory (SA), unsatisfactory (US) or not-assessable (NA), and the main reasons for concerns regarding study reliability also listed. All studies were of cross-sectional design.

NR = not reported; U = urban; R = rural; GP = general practitioner

**Table E - Studies related to trialled interventions**

| **Ref. (year of publication)** | **Study dates, design & location** | **Sample size & characteristics** | | **Intervention** | **Methods (M) & follow-up (F)** | **Main outcome measures (bold) & results** | **Quality assessment (numerical ratings and main concerns)** |
| --- | --- | --- | --- | --- | --- | --- | --- |
| **Article 2f: Education, communication, training and public awareness (school-based interventions)** | | | | | | | |
| s26 (2012) | 2004–2006; mediation analysis of RCT; Delhi & Chennai (U) | Project director, scientist, research assistant & eight community coordinators; age, sex NR | | Project MYTRI  (see Table 6) | M: data collected via interview, coded & used to generate mediation model mapping actions & processes; F: data collected 1.5 – 2 years post-start of intervention | **Central outcomes** identified as: 'not using tobacco' & 'advocating against tobacco use'. **Important mediators between intervention & outcomes**: knowledge skills, beliefs about tobacco, intentional, advocacy & self-efficacy beliefs | SA: 1,10,11; US: 2,4,8; NA: 3, 5-7, 9 (Aims unclear, small n-number, diverse subjects, potential for recall bias, assessor training unclear, analysis only partially described, unable to gauge extent of effects) |
| **Article 2f: Education, communication, training and public awareness (community-based intervention)** | | | | | | | |
| s27 (1995) | 1986–1992; QE; Karnataka (R) | n = between 3531 & 11401 per area at each follow-up; tobacco users; age NR; approx. 50 % male | | Health education once/week; group discussions once/month | M: 1 experimental, 2 control areas; intervention ‘lasted about 3 years’; F: survey at baseline, & 2- & 5- years post-start of intervention | Greater reduction **in tobacco use** in experimental cf. control areas (p < 0.0001); with intervention: **rates of tobacco use** dropped by 10.2 % (males), 16.3 % (females); **quit rates**: 26.5 % (males), 36.7 % (females) at 5 years | SA: 1; US: 2, 3, 7- 11; NA: 4-6 (Unclear why two control groups, recruitment, attrition & intended analysis unclear, baseline differences in tobacco use) |
| **Article 14: Demand reduction measures concerning tobacco dependence and cessation (pharmacological intervention)** | | | | | | | |
| s28 (2007) | 2001–04; QE; Delhi (U) | Group A/B: n = 372/87; mean age 37.6+ 13.8/39.2+ 13.5 years; 97.6 %/100 % male; cessation clinic attendees | | Group A: counseling; Group B: counselling & bupropion | M: Assigned to group A if Fagerstrom score 0–4, group B if > 5; F: intervention 7 weeks; follow-up at weeks 1-7, monthly for 3 months, & at months 6, 9 & 12; abstinence monitored via self-report & CO levels; reduction in intake measured at unspecified time point | Group A: 17.2 % **quit tobacco**, 8.9 % **reduced consumption**, 12.1 % no change; Group B: 59.8 % **quit**, 22.9 % **reduced**, 17.2 % no change; insomnia and dry mouth experienced in 2 & 4 subjects, respectively  **Continuous abstinence rates** S higher in Group B (p< 0.001 at 1, 3, 6 & 12 months) | SA: 1,2,6; US: 3,8,10,11; NA: 4,5,7,9 (Grouping according to addiction score, recruitment & data collection unclear, attrition bias relatively likely) |
| **Article 14: Demand reduction measures concerning tobacco dependence and cessation (non-pharmacological interventions)** | | | | | | | |
| s29 (2013) | Dates NR; RCT; Pune (U) | n = 47 controls, 50 individual counselling, 53 group counselling; industrial workers; tobacco users; motivated to quit; > 18 years; sex NR | | Controls: 10–15 minutes brief advice; Individual counselling: 5 sessions plus telephone counselling if required; Group counselling: 5 sessions | M: RCT, counselling sessions delivered over 6 months, self-reported quit rates used for analysis; cotinine assay used to validate quit reports at 6 months; F: Quit rates assessed at 3 & 6 months – unclear if all groups assessed at same time points | **Quit rates**: 0 % in control group, 6 % in individual counselling group, 7.55 % in group counselling group | SA: 1,2, 5; US: 6,7,9-11; NA: 3,4,8 (Small n-numbers, recruitment, data collection & intended analysis unclear, possible baseline group differences, additional results reported, main outcomes not tested for significance) |
| s30 (2012) | Dates NR; QE; location NR (U/R NR) | n = 224; tobacco users; < 35 to > 45 years; 1.8 % female; factory workers | | Cessation programme: individual & group therapeutic strategies | M: 3 contact intervention, 3 months between sessions; F: survey at end of each session (i.e. up to 6 months from start of intervention) | Post-end of 3 interventions: 17 % **quit tobacco use**; those with pre-cancerous lesions more likely to quit (OR 2.7 (95 % CI: 1.2 - 6.05)); more progression from pre-contemplative towards preparative **stage of change**, with each intervention | SA: 1; US: 2, 3, 6, 7,9-11; NA: 4, 5, 8 (No controls, risk of selection bias, potential attrition bias, methods & analysis unclear, additional outcomes reported, some reporting errors/inconsistencies) |
| s31 (2010) | Dates NR; QE; location NR (R) | n = 320; village residents; 14–37 years; tobacco users; sex NR | | Counselling regarding tobacco consumption | M: sessions delivered by trained NGO volunteers & traditional healers; F: data collection methods & timing of follow-up unclear | 289 **positive attitude to quitting**, 250 **quit**, 39 **abstained for short time** only | SA: n/a; US: 1,2; NA: 3-11 (Abstract; little information regarding sampling, intervention, intended analysis & results) |
| s32 (2010) | Dates NR; QE; location NR (R) | n = unclear; mean age 4 years; tobacco users; sex NR | | 3 counselling sessions/week | M: ‘subject evaluation’ carried out bi-monthly; objectives & methods of evaluation unclear; F: duration unclear | **Previous quit attempt**: 78 %; **dependence** 73 %; **quit-success rate** 22 % in < 35 years, 40 % in > 65 years; 30 % **stopped smoking**, **sustained quit rates** increased from 16 to 41 % during 1st year of programme | SA: n/a; US: 1,2; NA: 3-11 (Abstract; n-number, sampling, intervention, analysis & outcomes unclear; mean age 4 years, conclusions do not follow directly from results) |
| s33 (2009) | Dates NR; QE; location NR (R) | n = 378; 14–35 years; tobacco users; sex NR | | Counselling regarding tobacco consumption | M: 4 counselling sessions provided over 2 months by trained NGO volunteers & traditional healers; F: 24 months | 321 **‘positive attitude towards quitting’**, **284 quit tobacco for > 8 weeks**, 39 for 4 weeks then restarted | SA: n/a; US: 1,2; NA: 3-11 (Abstract; limited information regarding sampling, intervention, analysis & outcomes) |
| s34 (2007) | 2004–2005; interventional study; Varanasi (U) | n = 1200; university students; current smokers; 17–32 years; 100 % male | | Cessation counselling session; 'seven steps' method explained | M: unclear; F: unclear | At end of study: outcomes monitored not clearly described, but **quit rates** of 65 % | SA: 1; US: 2, 7, 9-11; NA: 3-6, 8 (Aims, methods & analysis unclear, no controls) |
| **Complex interventions addressing more than one FCTC article** | | | | | | | |
| s35 (2009) | Dates unclear; QE; Maharashtra (R) | | n = 50; tobacco users; age < 30 to> 50 years; sex NR; chemical industry employees | Lectures, oral examination, FGDs, counselling, community activities, pharmacotherapy 'if needed' | M: interventional cohort study; lectures/FGDs at 6-8 week intervals over 1 year; bupropion from 5^th^ session ‘if needed’; F: cessation monitored by self-reporting, cotinine test at final session | **Quit rates**: 40 % at end of study, 14.29 % in smokers, 51.52 % in smokeless users, 20 % in combination users; 5 users **offered bupropion**: one quit, 2 non-adherent due to side effects | SA: 1,2,4; US: 7, 9-11; NA: 3, 5, 6, 8 (Aims, recruitment, data collection and analysis unclear, additional outcomes reported, no controls) |
| s36 (2003) | 2002–2004; QE; Vaishali, Bihar (U/R NR) | | Residents of study area (population 1000000); age & sex NR | Individual counselling, group meetings, written materials, school programme, lobbying, wall paintings, health camps, alternative crop initiative | M: n = 900000 (minimal intervention), 100000 (intensive intervention); facilitators stationed in area for 5 months, then gradually withdrawn; F: unclear | Assessment intentions unclear  None rigorously measured | SA: n/a; US: 2, 10, 11; NA: 1, 3-9 (Interventions, recruitment & analysis unclear, few outcomes reported; difference between types of intervention unclear) |
| s37 (2004) | 2002; QE; Vaishali, Bihar (U/R NR) | | n = 824 ('intensive' intervention), 6020 ('minimal' intervention); age & sex NR; tobacco use: 51.4 – 55.3 % | (a) minimal: individual counselling, group & mass media interventions; wall paintings; (b) intensive: as (a) + school programme & health camps | M: area assigned intervention (a) or (b) on basis of criteria relating to existing tobacco control measures; F: unclear, but quit rates monitored for at least 12 months post-intervention | **Quit attempt:** 7.8 % of minimal intervention group, 27.4 % of intensive group; Post intervention: reduction in: gutka **sale, display & promotion**, **use by adolescents, use in schools & healthcare settings, & use in public** | SA: 1; US: 4, 9-11; NA: 2, 3, 5-8 (Selection & group assignment criteria-based, but unclear; data collection & intended analysis unclear, comparisons not tested, conclusions do not follow directly from results) |
| s38 (1990) | Dates unclear; prospective intervention study; Gujarat, Kerala, Andhra Pradesh (R) | | n = 36000 tobacco users (12000 in each of 3 districts); > 15 years; sex NR | Personal communication, films, posters, folk-drama, radio shows, cessation camps, dental clinics, newspaper articles | M: intervention applied in 3 areas; ‘oral exam, interview & intervention’ at baseline & follow-up; F: baseline survey & 10 annual follow-ups | **Quit rates** at end of 1, 5, 8 & 10 years **reported for 1 area only**: in Ernakulam (Kerala): 2 % quit at 1 year, 9.4 % at 5, 12.3 % at 8, & 14 % at 10 years | SA: n/a; US: 7, 9-11; NA: 1- 6, 8 (Aims, recruitment, data collection & analysis unclear, data only reported for one area, co-intervention relatively likely (10 year study)) |
| s39 (1986) | 1977 – 1983; QE; Kerala (K), Andhra Pradesh (A), Gujarat (G), (R) | | n = 36471 at baseline; tobacco users; > 15 years; Gujarat: 100 % male | Counselling after dental examination, films, posters, radio broadcasts, newspaper articles | M: One experimental & one control cohort selected from each district; interview & oral examination at each follow-up; F: controls: baseline 1966–67, follow-up at year 3, then annually until 10th year (results only reported to year 5); experimental cohort: baseline 1977–1983, annual follow-ups to 5^th^ year reported | **'Stoppage'**: K: 3 % controls, 9 % intervention; A: 5 % controls, 17 % intervention; G: 9 % controls, 13 % intervention; **'reduction'**: K: 9 % v 28 %, A: 8 % v 49 %; G: 21 % v 20 %; **5-year age-adjusted incidence of leukoplakia** (/1000): K: 47.8 v 11.4 (men), 33 v 5.8 (women) | SA: 1,7; US: 4, 5, 9-11; NA: 2, 3, 6, 8 (Aims and recruitment unclear, control & intervention groups studied over different periods, control groups appear to have experienced part of intervention, no statistical comparisons) |

Reviewed studies of trialled interventions excluded from analysis of outcomes, by FCTC Article. The numbers following the different quality ‘levels’ (SA, US, NA) indicate the aspect of quality assessment (see Box 1), rated as satisfactory (SA), unsatisfactory (US) or not-assessable (NA), and the main reasons for concerns regarding study reliability also listed.

RCT = randomised controlled trial; U = urban; R = rural; QE = quasi-experimental study; CO = carbon monoxide; NS = non-significant; S = significant; NR = not reported; OR = odds ratio; CI = confidence interval; NGO = non-governmental organisation; FGD = focus group discussion

**Supplementary references**

1. Dongre A, Deshmukh P, Murali N, Garg B. Tobacco consumption among adolescents in rural India: where and how tobacco control should focus its attention? Indian J Cancer 2008;45: 100–106.
2. Arora M, Tewari A, Tripathy V, Nazar GP, Juneja NS, Ramakrishnan L, et al. **Community-based model for preventing tobacco use among disadvantaged adolescents in urban slums of India.** Health Promot Int. 2010;25: 143–152
3. Panda B, Rout A, Pati S, Chauhan AS, Tripathy A, Shrivastava R, et al. Tobacco control law enforcement and compliance in Odisha, India – implications for tobacco control policy and practice. Asian Pacific J Cancer Prev 2012;13: 4631-4637.
4. Schensul JJ, Nair S, Bilgi S, Cromley E, Kadam V, Mello SD, et al. Availability, accessibility and promotion of smokeless tobacco in a low-income area of Mumbai. Tob Control 2013;22: 5 324-330.
5. Arora M, Tewari A, Nazar GP, Gupta VK, Shrivastava R. Ineffective pictorial health warnings on tobacco products: Lessons learnt from India. Indian J Pub Health 2012;56: 61.
6. Oswal KC, Raute LJ, Pednekar MS, Gupta PC. Are Current Tobacco Pictorial Warnings in India Effective? Asian Pac J Cancer Prev 2011;12: 121-124
7. Raute LJ, Pednekar MS, Gupta PC. Pictorial health warnings on cigarette packs: A population based study findings from India. Tob Use Insights 2009;2: 11–16.
8. Gunaseelan R, Shanthi S, Sowmya R, Datta M. Areca nut use among rural residents of Sriperambudur Taluk: a qualitative study. Indian J Dent Res 2007;18: 11-14
9. Mohan S, Mini GK, Thankappan KR. High knowledge of Framework Convention on Tobacco Control provisions among local government representatives does not translate into effective implementation: Findings from Kerala, India. Public Health 2013;127: 178-181
10. Surani NS, Shroff HP. Mass media exposure to tobacco messages among secondary school children in Mumbai. Indian J Public Health 2012;56: 159-162.
11. Singh V, Gupta R. Prevalence Of Tobacco Use And Awareness Of Risks Among School Children In Jaipur. J Assoc Physicians India 2006;54: 609-612.
12. Raute LJ, Gupta PC, Pednekar MS. Smoking ban and indoor air quality in restaurants in Mumbai, India. Indian J Occup Environ Med 2011;15: 68-72.
13. Kaur J, Prasad VM. Air nicotine monitoring for second hand smoke exposure in public places in India. Indian J Community Med 2011;36: 98-103.
14. Lal PG, Wilson NC, Singh RJ. Compliance surveys: an effective tool to validate smoke-free public places in four jurisdictions in India. Int J Tuberc Lung Dis 2011;15: 565-566.
15. Bhatia V, Puri S, Kaur A, Mayank V. Impact of ban of smoking in first smoke free city of Chandigarh in India. 14^th^ World Conference on Tobacco or Health. Mumbai, India, 2009.
16. Mishra A, Arora A, Stigler MH, Komro KA, Lytle LA, Reddy KS, et al. Indian youth speak about tobacco: results of focus group discussions with school students. Health Educ Behav 2005;32: 363-79.
17. Sinha DN, Gupta PC, Warren CW, Asma S. Effect of school policy on tobacco use by school personnel in Bihar, India. J Sch Health 2004;74 : 3-5.
18. Sinha DN, Gupta PC, Warren CW, Asma S. School policy and tobacco use by students in Bihar, India. Indian J Public Health 2004;48: 118–122.
19. Sorensen G, Gupta PC, Sinha DN, Shastri S, Kamat M, Pednekar MS, et al. Teacher tobacco use and tobacco use prevention in two regions in India: qualitative research findings. Prev Med. 2005;41:424-432.
20. Sharma I, Sarma PS, Thankappan KR. Awareness, attitude and perceived barriers regarding implementation of the Cigarettes and Other Tobacco Products Act in Assam, India. Ind J Cancer 2010;47:63-68.
21. Mishra GA, Shastri SS, Uplap PA, Majmudar PV, Rane PS, Gupta SD. Establishing a model workplace tobacco cessation program in India. Indian J Occup Environ Med 2009;13: 97-103.
22. Anwar S, Williams SA, Scott-Smith J, Sage H, Baweja S, Singal M, et al. A comparison of attitudes and practices of gutka users and non-users in Chitrakoot, India. A pilot. [Prim Dent Care](http://www.ncbi.nlm.nih.gov/pubmed/15703153) 2005;12: 5-10.
23. Saddichha S, Rakha DP, Patil BK, Murthy P, Benegal V, Isaac MK. Knowledge, attitude and practices of Indian dental surgeons towards tobacco control: advances towards prevention. Asian Pac J Cancer Prev 2010;11: 939-942.
24. Pati S. A study on tobacco cessation practice among general practitioners in Orissa (India). Lung Cancer 2005;49: S195.
25. Binnal A, Rajesh GR, Ahmed J, Denny C, Nayak SU. Insights into smoking and its cessation among current smokers in India. Asian Pacific J Cancer Prev 2013;14: 2811-2818.
26. Bate SL, Stigler MH, Thompson MS, MacKinnon DP, Arora M. A qualitative mediation study to evaluate a school-based tobacco prevention program in India (Project MYTRI). Field Methods 2012;24: 194-215.
27. Anantha N, Nandakumar A, Vishwanath N, Venkatesh T, Pallad YG, Manjunath P, et al. Efficacy of an anti-tobacco community education program in India. Cancer Causes Control 1995;6: 119-29.
28. Kumar R, Kushwah AS, Mahakud GC, Prakash S, Vijayan VK. Smoking cessation interventions and continuous abstinence rate at one year. Indian J Chest Dis Allied Sci 2007;49: 201-207.
29. Savant SC, Hegde-Shetiya S, Agarwal D, Shirhatti R, Shetty D. Effectiveness of individual and group counselling for cessation of tobacco habit amongst industrial workers in Pimpri, Pune – An interventional study. Asian Pacific J Cancer Prev 2013;14: 1133-1139.
30. Pimple S, Pednekar M, Mazumdar P, Goswami S, Shastri S. Predictors of quitting tobacco--results of a worksite tobacco cessation service program among factory workers in Mumbai, India. Asian Pac J Cancer Prev 2012;13: 533-8.
31. Shankpal PD, Shankpal V. Quit-tobacco: A decade of absolute dedication in lung cancer prevention in Rural India. J Thorac Oncol 2010;5: S54.
32. Shankpal PD, Sankpal V. Abstinence and effectiveness of smoking cessation project: Experiences of Indian cancer NGO about predictable factors. J Thorac Oncol 2010;5: S48-S49.
33. Sankpal PD, Narayanan RS, Sankpal VP. QUIT-tobacco programs: Initiatives of community NGO to control respiratory cancer in tribal/rural India. Psycho-oncology 2009;18: S111-S112.
34. Srivastava GN. Seven steps of gradual cessation of smoking--an example from India. Vojnosanit Pregl 2007;64: 405-408.
35. Mishra GA, Majmudar PV, Gupta SD, Rane PS, Uplap PA, Shastri SS. Workplace tobacco cessation program in India: A success story. Indian J Occup Environ Med. 2009;13: 146-153.
36. Sinha ND, Singh S, Jha M, Singh M. Report on tobacco cessation through community intervention in India. New Delhi: WHO SEARO, 2003.
37. Sinha DN, Dobe M. Effectiveness of tobacco cessation intervention programs. Indian J Public Health 2004;48: 138-143.
38. Aghi MB, Gupta PC, Bhonsle RB, Murti PR. Communication strategies for intervening in the tobacco habits of rural populations in India. In: Gupta PC, Hamner JE III, Murti PR, editors. Control of tobacco-related cancers and other diseases. Proceedings of an International Symposium; January 15-19, 1990; TIFR, Mumbai, India. Mumbai: Oxford University Press; 1992 p. 303-306.
39. Gupta PC, Mehta FS, Pindborg JJ, Aghi MB, Bhonsle RB, Daftary DK, et al. Intervention study for primary prevention of oral cancer among 36 000 Indian tobacco users. Lancet 1986;1: 1235-9.
